# Novel FOXM1 inhibitor identified via gene network analysis induces autophagic FOXM1 degradation to overcome chemoresistance of human cancer cells

**DOI:** 10.1038/s41419-021-03978-0

**Published:** 2021-07-14

**Authors:** Mikhail S. Chesnokov, Marianna Halasi, Soheila Borhani, Zarema Arbieva, Binal N. Shah, Rick Oerlemans, Irum Khan, Carlos J. Camacho, Andrei L. Gartel

**Affiliations:** 1 University of Illinois at Chicago, Department of Medicine, Chicago, IL USA; 2 Massachusetts General Hospital, Department of Surgery, Boston, MA USA; 3 University of Illinois at Chicago, Genome Research Core, Chicago, IL USA; 4 University of Pittsburgh, College of Medicine, Pittsburgh, PA USA

**Keywords:** Oncogenes, Target identification

## Abstract

FOXM1 transcription factor is an oncogene and a master regulator of chemoresistance in multiple cancers. Pharmacological inhibition of FOXM1 is a promising approach but has proven to be challenging. We performed a network-centric transcriptomic analysis to identify a novel compound STL427944 that selectively suppresses FOXM1 by inducing the relocalization of nuclear FOXM1 protein to the cytoplasm and promoting its subsequent degradation by autophagosomes. Human cancer cells treated with STL427944 exhibit increased sensitivity to cytotoxic effects of conventional chemotherapeutic treatments (platinum-based agents, 5-fluorouracil, and taxanes). RNA-seq analysis of STL427944-induced gene expression changes revealed prominent suppression of gene signatures characteristic for FOXM1 and its downstream targets but no significant changes in other important regulatory pathways, thereby suggesting high selectivity of STL427944 toward the FOXM1 pathway. Collectively, the novel autophagy-dependent mode of FOXM1 suppression by STL427944 validates a unique pathway to overcome tumor chemoresistance and improve the efficacy of treatment with conventional cancer drugs.

## Introduction

Forkhead box (FOX) protein M1 (FOXM1) is a transcription factor with pronounced pro-oncogenic functions [1, 2]. It is overexpressed in the majority of human cancers and impacts all hallmark tumor aspects, including proliferation, survival, metastasis, inflammation, angiogenesis, and treatment resistance [3–5]. Due to this, FOXM1 serves as a crucial regulator of tumor development, and its overexpression portends a poor prognosis for patients, promoting aggressive tumor phenotype and high resistance to current therapeutic approaches [3, 5].

Inherent or acquired chemoresistance remains the major contributor to cancer therapy failure. While a multitude of molecular mechanisms can underlie chemoresistance development [6], FOXM1 is repeatedly identified as a common element associated with weaker response to conventional chemotherapeutic agents in various tumors [7–10]. FOXM1 reduces the efficacy of platinum-based drugs through an increase in DNA damage repair [11, 12], oxidative stress prevention [13], and drug efflux [14]. It also contributes to 5-fluorouracil (5-FU) resistance by promoting ABCC10 transporter expression [8] or causing overexpression of thymidylate synthase, the primary 5-FU target [15]. Moreover, it is involved in taxane resistance through regulation of JNK/mitochondrial signaling [16], AMPK/mTOR-mediated autophagy [9], or microtubule dynamics [17]. Accordingly, knockdown of FOXM1 or its downstream targets increases the sensitivity to standard chemotherapy in many human cancers, including colorectal [8, 15], gastric [18], lung [19], ovarian cancer [20], retinoblastoma [14], and nasopharyngeal carcinoma [21]. Therefore, inhibition of FOXM1 may prove critical for developing effective therapeutic solutions for cancer chemoresistance problem.

Inhibition of pro-oncogenic regulators with small molecules is a popular and established approach in current clinical practice. However, targeting of transcription factors has been particularly challenging. A growing number of direct and indirect pharmacological FOXM1 inhibitors have been identified, including thiostrepton [22], honokiol [23], bortezomib [24], siomycin A [25], curcumin [26], SR-T100 [27], FDI-6 [28], RCM-1 [29], and DFS lignan [30]. FOXM1 inhibition efficiently sensitizes cancer cells to conventional chemotherapy, yet the often unknown inhibitory pathways of these compounds or their off-target actions exhibit undesired secondary effects like general proteasome inhibition or possible activity toward other targets, especially other FOX proteins. Therefore, there is an urgent need to develop efficient and selective agents with a clear mode of action against FOXM1 activity.

Here we use a gene network analysis approach to discover a novel small molecule STL427944 that selectively targets FOXM1 pathway. This compound suppresses FOXM1 protein through a novel two-step mechanism that includes translocation of nuclear FOXM1 protein to the cytoplasm and its subsequent autophagic degradation. STL427944 treatment results in sensitization of cancer cells to multiple chemotherapeutic agents. We also provide transcriptome-supported evidence that STL427944 exhibits selectivity toward suppressing FOXM1-controlled regulatory pathways. The unique mode of action revealed by our studies, which, unlike previously reported [31], does not respond to proteasome inhibitors, establishes a novel pathway to target this master regulator of chemoresistance in multiple cancers.

## Results

### Transcriptomic analysis identifies small molecules disrupting FOXM1 pathway

The development of pharmaceutical agents inhibiting pro-oncogenic proteins is a major area in cancer treatment research. Historically, these studies have been conducted in a target-centric way, focusing on molecules directly interacting with a protein of interest. However, this requirement for direct binding significantly limits the number of options, while usage of a single target for the initial screening increases the chances of identifying agents with unwanted nonspecific effects. Recently, Pabon et al. [32] adopted a different, network-centric strategy. Transcriptomic and proteomic data were used to identify agents affecting specific disease pathways, with the goal of revealing novel targets leading to specific inactivation of the whole pro-oncogenic pathway. This approach leverages the whole network of protein interactions that could impact the protein of interest by either direct binding to it or indirect binding to a member of the network [33].

We applied this new network-based screening concept to identify potential small molecule inhibitors of the FOXM1 pathway activity, using differentially expressed (DE) gene signatures from the National Institutes of Health’s Library of Integrated Network-Based Cellular Signatures (LINCS) L1000 dataset. Unfortunately, LINCS database does not contain datasets describing transcriptomic effects of *FOXM1* knockdown. However, our previous findings demonstrated that FOXM1 activity and protein level are strongly dependent on its interaction with nucleophosmin (NPM) [34]. We therefore compared transcriptional profiles between knockdowns of *NPM1* gene (as a proxy to *FOXM1* knockdown) and responses of the same cell types to thousands of distinct bioactive compounds. This screen resulted in 264 compounds that showed either a direct correlation with the *NPM1* knockdown or an indirect correlation with the knockdown of an NPM-binding partner (for additional details, see [33]). Furthermore, excluding kinase inhibitors and compounds not available for purchase, we compared the profiles of these hits with the changes in expression of established FOXM1 targets [35, 36] present in our dataset across seven cancer cell lines (A549, MCF7, VCAP, HA1E, A375, HCC515, and HT29). As expected, all eight genes are downregulated by *NPM1* knockdown or knockdowns of major FOXM1 targets *AURKB* and *MYC* (Table 1). This analysis highlighted STL427944 (C_25_H_23_N_7_O_4_, PubChem CID 9592990) and benzamil (C_13_H_15_C_l2_N_7_O, PubChem CID 108107), two top hits predicted to disrupt NPM–FOXM1 gene network. Consensus changes in FOXM1 targets expression suggested that STL427944 should be a more potent and universal inhibitor of FOXM1-dependent network than benzamil (Table 1), therefore, we proceeded with experimental characterization of STL427944 (henceforth referred to as “STL”).

**Table 1:** Changes in expression of FOXM1-associated genes across a set of human cancer cell lines (LINCS data) caused by *NPM1*, *AURKB*, and *MYC* knockdown or treatment with candidate FOXM1-inhibiting agents.

GeneID	Gene symbol	Consensus gene expression changes (z-score values)				
		*NPM1* knockdown	*AURKB* knockdown	*MYC* knockdown	STL427944 treatment	Benzamil treatment
Direct target genes activated by FOXM1						
332	*BIRC5*	−1.44	−1.43	−15.00	−6.05	−0.04
891	*CCNB1*	−3.21	−1.64	−7.87	−1.65	−1.30
9133	*CCNB2*	−2.55	−1.92	−16.66	−2.05	−0.90
983	*CDK1*	−3.32	−2.18	−7.66	−2.79	0.29
991	*CDC20*	−3.79	−3.26	−24.50	−9.48	−1.12
993	*CDC25A*	−1.19	−2.19	−7.96	−6.81	0.16
5347	*PLK1*	−1.39	−2.30	−10.60	−4.39	0.05
11065	*UBE2C*	−3.01	−1.27	−12.57	−4.05	−0.19

### STL treatment suppresses FOXM1 protein levels in human cancer cells

**Fig. 1:**
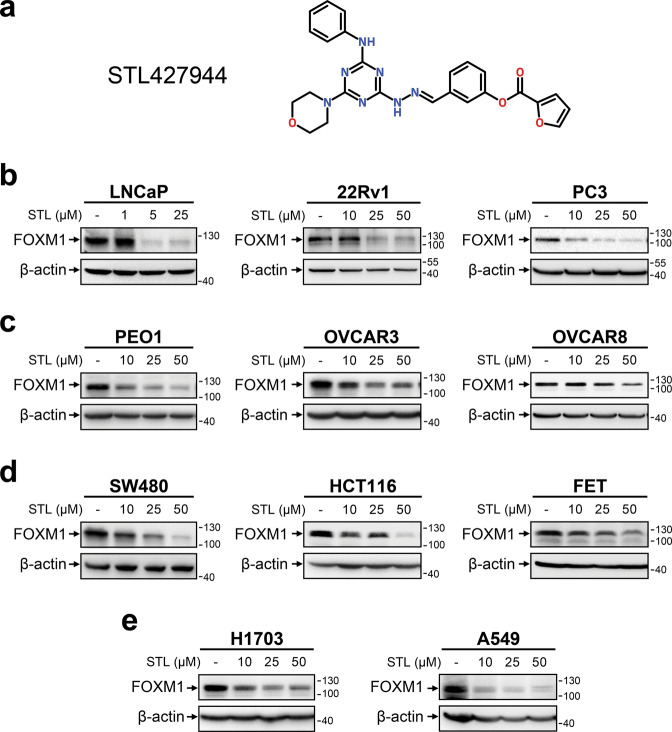
STL treatment causes dose-dependent suppression of FOXM1 protein levels in cancer cell lines of different etiology. **a** Structural formula of STL. **b-e** Various cell lines representing human prostate (**b**), HGSOC (**c**), colorectal (**d**), or NSCLC (**e**) cancer were treated with increasing concentrations of STL for 24 h. Total protein samples obtained from treated cells were analyzed for FOXM1 protein levels via immunoblotting, β-actin was used as internal loading control.

**Fig. 2:**
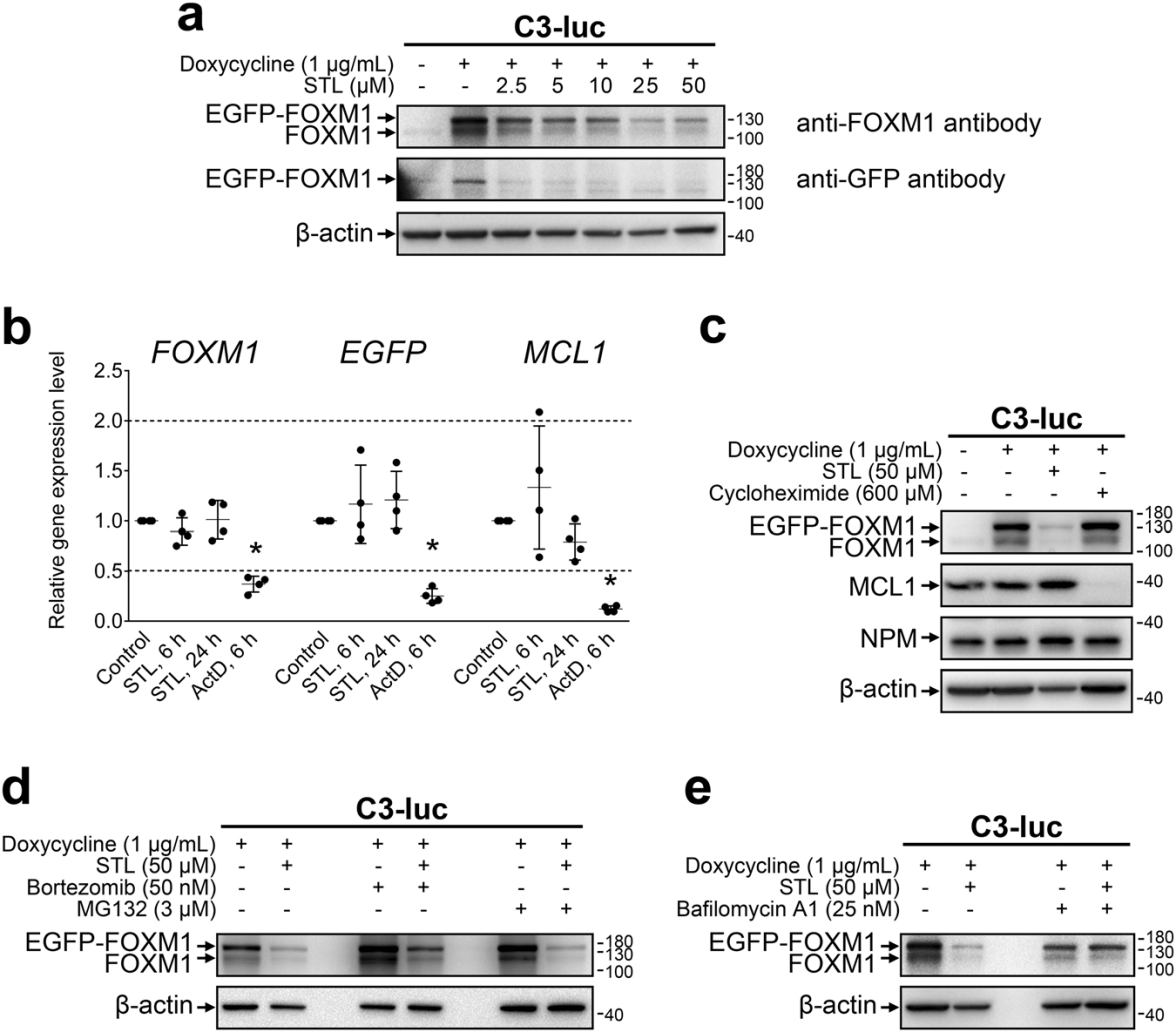
STL inhibits FOXM1 expression on protein level via autophagy-dependent mechanism. **a** C3-luc cells stimulated with doxycycline to induce expression of EGFP-FOXM1 fusion protein were treated with increasing concentrations of STL for 24 h. Total protein samples were analyzed via immunoblotting for FOXM1 and GFP expression, β-actin was used as an internal loading control. **b** Doxycycline-stimulated C3-luc cells were treated with 50 μM STL for 6 or 24 h and 10 μg/mL ActD for 6 h. Total RNA samples were analyzed for *FOXM1*, *GFP*, and *MCL1* transcript levels via RT-qPCR, 18 S rRNA was used as a reference transcript. Data are presented as means ± S.D. and individual datapoints, *N* = 4, * – exact *p* = 0.02857 (Mann–Whitney U test, two-tailed). **c** C3-luc cells were treated with indicated concentrations of doxycycline, STL, or CHX for 24 h. Total protein samples were analyzed via immunoblotting for FOXM1, NPM, and MCL1 expression, β-actin was used as an internal loading control. **d** Doxycycline-stimulated C3-luc cells were treated with STL for 24 h in the presence of bortezomib or MG132. Total protein samples were analyzed via immunoblotting for FOXM1 expression, β-actin was used as an internal loading control. **e** Doxycycline-stimulated C3-luc cells were treated with STL for 24 h in the presence of bafilomycin A1. Total protein samples were analyzed via immunoblotting for FOXM1 expression, β-actin was used as an internal loading control.

To experimentally confirm FOXM1-suppressing effect of STL, we used human cancer cell lines of different origin (Supplementary Table 1). Treatment with STL resulted in dose-dependent reduction of FOXM1 protein levels in all examined cell lines (Figs. 1, 2a). Prominent FOXM1 suppression was often achieved with STL concentrations as low as 5–10 μM (LNCaP, PC3, and A549 cells) with maximum efficiency reached at 25–50 μM. Since FOX proteins display significant structural homology [37], we evaluated FOXO1 and FOXO3A levels (Supplementary Fig. 1) and confirmed that STL does not suppress FOX proteins in general.

### STL targets FOXM1 protein to lysosome-mediated degradation

FOXM1 inhibitors may exert their action at multiple levels, including transcriptional, translational, and post-translational effects. To further investigate how STL suppresses FOXM1, we utilized an experimental model based on U2OS human osteosarcoma cells previously described as C3-luc [25] that express EGFP-FOXM1 fusion protein controlled by doxycycline-inducible promoter. Due to the positive autoregulatory loop [38], exogenous EGFP-FOXM1 protein also promotes the expression of endogenous FOXM1. Treatment of doxycycline-stimulated C3-luc cells with STL drastically decreased the levels of both endogenous and exogenous FOXM1 in a dose-dependent manner starting with 2.5 μM (Fig. 2a). To exclude possible STL effects on FOXM1 recognition by the antibody, the same samples were additionally probed for GFP levels with a similar result (Fig. 2a). The ability to suppress EGFP-FOXM1 expression driven by doxycycline-controlled promoter indicates that STL effect on FOXM1 is not dependent on any signaling pathway regulating the activity of endogenous *FOXM1* promoter. We therefore evaluated possible effects of STL upon FOXM1 mRNA stability and translation efficiency.

To investigate mRNA-related effects of STL, we evaluated the levels of all FOXM1, exogenous EGFP-FOXM1 only, and short-lived *MCL1* transcripts after treatment with STL or general transcription inhibitor actinomycin D (ActD). While ActD prominently reduced the levels of inspected genes, STL treatment did not significantly affect them (Fig. 2b, Supplementary Fig. 2), ruling out the possibilities of STL acting as a global transcription inhibitor or inducing prominent FOXM1 mRNA degradation. At the same time, STL reduced the expression of FOXM1 target gene *AURKB* in a dose-dependent manner, indicating functional FOXM1 inactivation (Supplementary Fig. 2). STL treatment also did not affect the level of short-lived MCL1 protein that was very sensitive to general translation inhibition by cycloheximide (CHX, Fig. 2c). Surprisingly, STL addition to cells already being treated with CHX did not cause FOXM1 repression. We therefore evaluated the kinetics of FOXM1 protein levels after addition of STL, CHX, or both, and confirmed that only STL alone was causing significant FOXM1-level reduction over time (Supplementary Fig. 3). FOXM1 level in cells treated simultaneously with STL and CHX showed quick decrease but then remained stable for 24 h. These results indicate that FOXM1 is suppressed by STL at the post-translational stage, most likely through increased protein degradation that may be mediated via short-lived proteins.

Since STL treatment partially replicates transcriptomic effects of *NPM1* knockdown, we additionally tested if STL could reduce FOXM1 level through NPM suppression [34]. However, treatment with the highest STL concentration did not affect NPM protein levels in C3-luc cells (Fig. 2c), supporting the hypothesis of STL selectivity toward FOXM1.

In general, intracellular proteins are degraded via two mechanisms: ubiquitin–proteasome pathway or autophagy. Proteasome inhibition by bortezomib or MG132 did not rescue FOXM1 from suppression by STL (Fig. 2d). On the other hand, inhibition of lysosome function by bafilomycin A1 (BafA1) completely prevented STL-dependent reduction of FOXM1 level (Fig. 2e), suggesting that STL facilitates lysosome-mediated destruction of FOXM1 protein. However, BafA1 treatment itself caused prominent cell stress (based on cell morphology, results not shown) and reduced the initial FOXM1 level independently of STL. We therefore performed a more detailed study of STL effects on autophagy and lysosomes.

### STL relocalizes FOXM1 to the cytoplasm and promotes its autophagic degradation

**Fig. 3:**
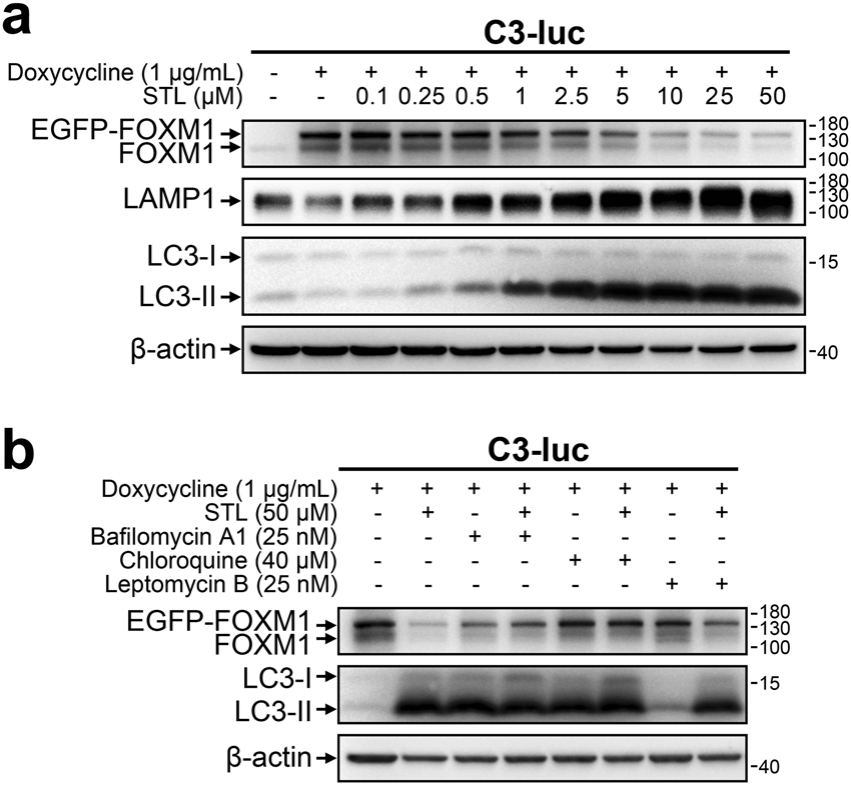
STL-dependent FOXM1 degradation is autophagy-mediated and is prevented by nuclear-export arrest. **a** C3-luc cells were treated with indicated concentrations of doxycycline and STL for 24 h. Total protein samples were analyzed via immunoblotting for FOXM1, LAMP1, and LC3 expression, β-actin was used as an internal loading control. **b** C3-luc cells were treated with indicated concentrations of doxycycline, STL, bafilomycin A1, CQ, and LMB for 24 h. Total protein samples were analyzed via immunoblotting for FOXM1 and LC3 expression, β-actin was used as an internal loading control.

Autophagic lysosomal activity is considered to be relatively low under normal conditions. We hypothesized that prominent lysosome-dependent FOXM1 degradation caused by STL should be associated with autophagy induction. Indeed, treatment of C3-luc cells with 0.5 μM or higher concentrations of STL results in upregulation of both autophagy marker protein LC3-II and lysosomal membrane protein LAMP1 (Fig. 3a). The same effect was observed in OVCAR3 and HCT116 cells (Supplementary Fig. 4), confirming that STL can universally induce autophagy in mammalian cells. Accumulation of LC3-II and LAMP1 was evident in 4 h after treatment start and gradually progressed with time; the addition of CHX in combination with STL prevented LC3-II increase over time, while LAMP1 levels were slowly decreasing (Supplementary Fig. 3a). These results strongly support the idea of autophagy-dependent FOXM1 degradation because CHX is known to inhibit autophagosome maturation [39–41]. To perform a more specific test, we used chloroquine (CQ) that prevents autophagosome–lysosome fusion [42], inhibits autophagy without affecting lysosome-mediated degradation, and has less impact on initial FOXM1 levels in C3-luc cells. Addition of CQ completely rescued FOXM1 protein levels from suppression by STL (Fig. 3b) and thereby determined that autophagosome maturation into autolysosomes is essential for STL effect on FOXM1. Time-course evaluation of autophagic flux in C3-luc cells demonstrated that STL-dependent LC3-II accumulation occurs much faster than in the case of autophagosome degradation arrest by CQ, thereby confirming autophagy stimulation by STL (Supplementary Fig. 5).

While autophagosomes are cytoplasmic structures, FOXM1 is predominantly located in the nucleus [29]. It may undergo autophagic degradation either via nonspecific macronucleophagy [43] or after translocation to the cytoplasm. Arrest of nuclear protein export with leptomycin B (LMB) caused partial reversal of STL-induced FOXM1 suppression but did not prevent autophagy activation (Fig. 3b), suggesting that FOXM1 only becomes available to autophagosomes after its relocation to the cytoplasm. It also indicates that STL induces autophagy independently of FOXM1 suppression.

**Fig. 4:**
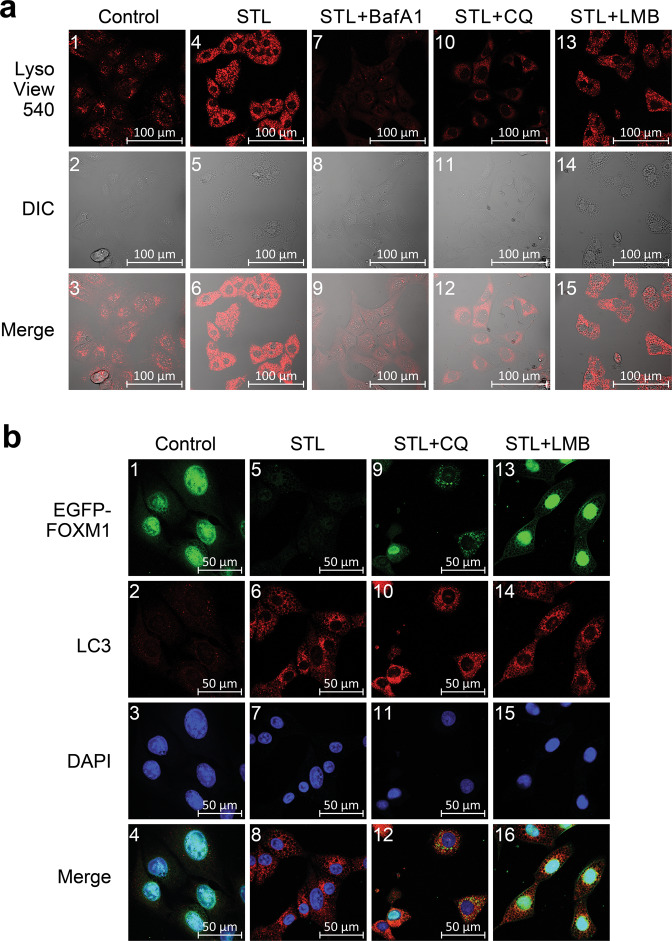
STL promotes active lysosome formation and FOXM1 translocation from the nucleus to the cytoplasmic autophagosomes. **a** Doxycycline**-**stimulated C3-luc cells expressing EGFP-FOXM1 fusion protein were treated with vehicle (“Control”, panels 1–3), 50 μM STL alone (“STL”, panels 4–6), or 50 μM STL in combinations with 25 nM BafA1 (“STL + BafA1”, panels 7–9), 40 μM CQ (“STL + CQ”, panels 10–12), and 25 nM LMB (“STL + LMB”, panels 13–15) for 12 h. Lysosomes were stained with vital LysoView 540 dye (red), cell morphology was analyzed using differential interference contrast (DIC) microscopy. **b** Doxycycline-stimulated C3-luc cells were treated with vehicle (“Control”, panels 1–4), 50 μM STL alone (“STL”, panels 5–8), or 50 μM STL in combinations with 40 μM CQ (“STL + CQ”, panels 9–12) and 25 nM LMB (“STL + LMB”, panels 13–16) for 24 h. Cells were stained for LC3 protein, nuclei were counterstained with DAPI. EGFP-FOXM1 (green), LC3 (red) and DAPI (blue) fluorescence was analyzed using confocal laser-scanning microscopy.

To investigate this hypothesis, we stained drug-treated C3-luc cells with vital lysosome-specific dye LisoView (Fig. 4a, Supplementary Fig. 6). Confocal microscopy demonstrated that STL induced prominent formation of acidic intracellular vesicles that are assumed to be lysosomes or autolysosomes. As expected, BafA1 completely prevented the formation of these vesicles, confirming their lysosomal nature; however, addition of CQ to STL also returned lysosomal staining to baseline level and drastically reduced the number of vesicles, indicating that they originate through autophagosome maturation. Moreover, LMB did not prevent autolysosome formation, additionally proving that FOXM1 relocalization to the cytoplasm is crucial for its STL-dependent degradation.

We further studied intracellular localization of FOXM1 and autophagosome marker LC3 using confocal microscopy (Fig. 4b, Supplementary Fig. 7). Doxycycline-stimulated C3-luc cells demonstrate clear nuclear localization of EGFP-FOXM1 protein and low background levels of LC3 in the cytoplasm. As expected, STL treatment caused drastic reduction of nuclear EGFP-FOXM1 signal and prominent LC3 staining in the cytoplasm, indicating autophagy induction; the pattern of LC3 staining suggests that it delineates the cytoplasmic vesicles. Cells treated with STL + CQ displayed a high fraction of cells with cytoplasmic localization of EGFP-FOXM1, where it colocalized with LC3-positive puncta that commonly correspond to autophagosomes. Addition of LMB to STL retained EGFP-FOXM1 in the nucleus and prevented its degradation without a visible effect upon accumulation of LC3-labeled autophagic vesicles in the cytoplasm (see Table 2 for colocalization test results). Taken together, these results imply that STL-dependent FOXM1 suppression is a two-step process: STL induces cytoplasmic autophagosome accumulation and then stimulates FOXM1 protein export to the cytoplasm, where it is transported to autophagosomes and subsequently destroyed. In the presence of CQ, FOXM1 is still transported to the cytoplasm, but accumulates in immature autophagosomes instead of degradation, resulting in the same FOXM1 levels detected in total cell protein samples (Figs. 3b, 4b). Nevertheless, FOXM1 sequestration in the cytoplasm should still functionally inactivate it. In agreement with this statement, we observed only a slight rescue of suppressed FOXM1 target genes in cells treated with CQ + STL combination, while LMB was able to return their expression back to baseline levels due to FOXM1 retention in the nuclei (Supplementary Fig. 8).

**Table 2:** Colocalization of EGFP-FOXM1 with cytoplasmic LC3-positive vesicles or DAPI-stained nuclear DNA (See Fig. 4b and Supplementary Fig. 7 for images).

Treatment	Manders’ Overlap Coefficient	
	FOXM1 with LC3	FOXM1 with nucleic DNA
Control	N/A	0.926 ± 0.043
50 μM STL	N/A	N/A
40 μM CQ	0.074 ± 0.038	0.946 ± 0.028
40 μM CQ + 50 μM STL	0.635 ± 0.218 (***)	0.281 ± 0.316 (***)
25 nM LMB	N/A	0.847 ± 0.07
25 nM LMB + 50 μM STL	0.243 ± 0.081 (^###^)	0.837 ± 0.043 (^###^)

Data are presented as Manders’ Overlap Coefficients ± S.D., *N* = 12, *** *p* < 0.001 in comparison to “40 μM CQ” sample, ^###^ *p* < 0.001 in comparison to “40 μM CQ + 50 μM STL” sample (Mann–Whitney U test, two-tailed). N/A – not applicable due to very low signal.

### STL-induced FOXM1 suppression sensitizes cancer cells to chemotherapeutic agents

Sensitization to antitumor drugs is the most well-characterized effect of FOXM1 downregulation in cancer cells. We therefore assumed that STL treatment should reduce chemoresistance as well and estimated the cytotoxic effects of STL alone or in combination with other agents. Considering that FOXM1 provides resistance to a broad spectrum of drugs, we used several agents with different mechanisms of action: direct DNA damage (carboplatin), DNA synthesis inhibition (5-FU), or cell division disruption (paclitaxel, docetaxel). Each drug was tested in model cell lines belonging to cancer type for which treatment with this particular drug was approved by FDA.

**Fig. 5:**
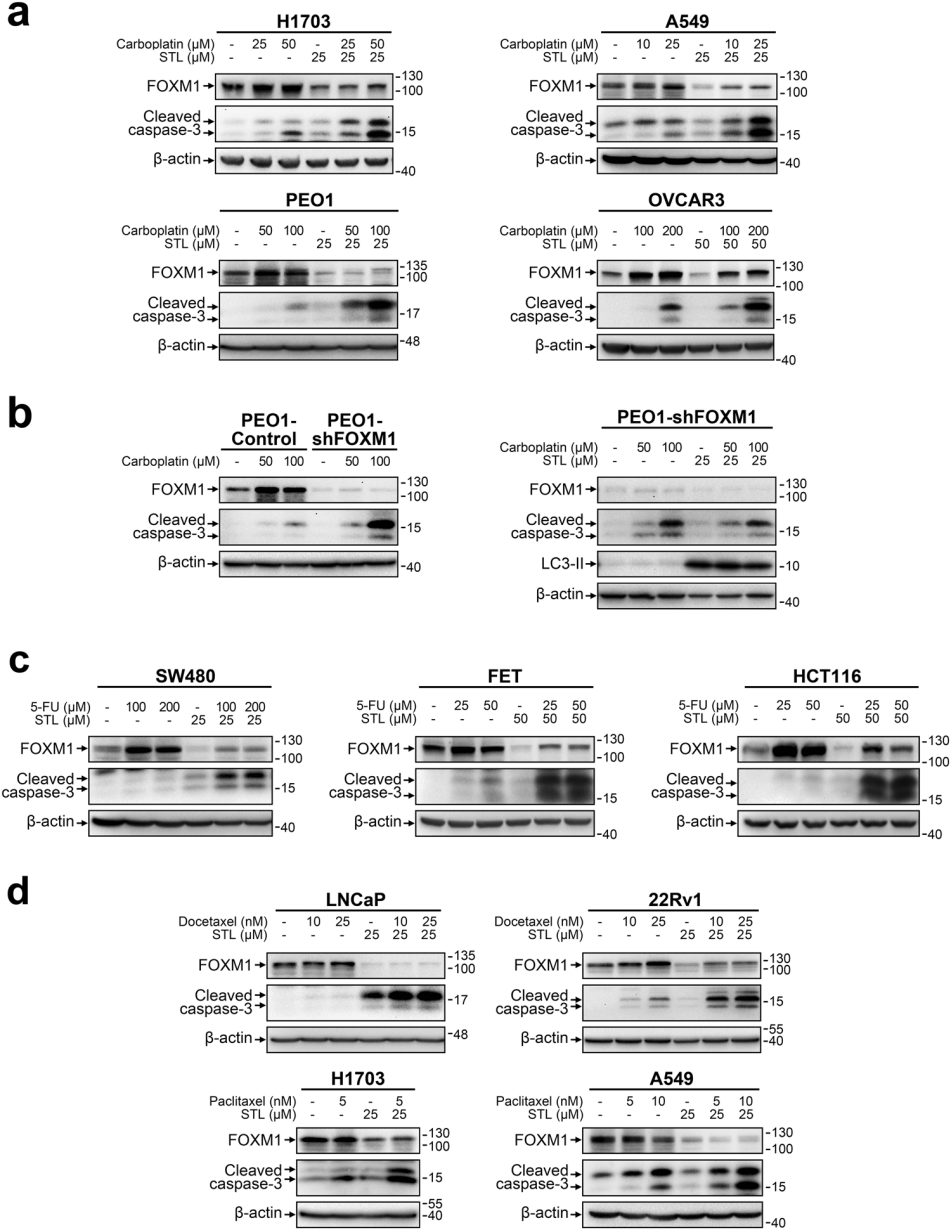
STL enhances the cytotoxic effect of conventional chemotherapeutic drugs through suppression of FOXM1. **a** NSCLC and HGSOC cell lines were treated with indicated concentrations of carboplatin and STL alone or in combination for 48 h. **b** PEO1 cells with stable FOXM1 knockdown were treated with indicated concentrations of carboplatin and STL alone or in combination for 48 h. **c** Colorectal cancer cell lines were treated with indicated concentrations of 5-FU and STL alone or in combination for 24 h. **d** Prostate cancer and NSCLC cell lines were treated with indicated concentrations of docetaxel, paclitaxel, and STL alone or in combination for 24 h. In all cases, total protein samples were obtained from cells immediately after treatment and analyzed for FOXM1, cleaved caspase-3 and LC3 levels via immunoblotting, β-actin was used as an internal loading control.

Lung cancer (H1703, A549) and ovarian cancer (PEO1, OVCAR3) cells treated with sublethal concentrations of carboplatin display a prominent increase in FOXM1 protein levels. Addition of STL in combination with carboplatin efficiently prevented FOXM1 activation (Fig. 5a), resulting in decreased (PEO1, H1703 and A549) or the same (OVCAR3) FOXM1 protein levels in comparison with the corresponding control samples. STL alone did not exert prominent cytotoxic effects, but cells treated with carboplatin+STL combination displayed a significant increase in cleaved caspase-3 level when compared with samples treated with carboplatin alone, indicating a strong synergistic pro-apoptotic effect between two drugs.

To test if STL can cause chemosensitization through other mechanisms besides FOXM1 suppression, we used PEO1 cells with stable shRNA-mediated FOXM1 knockdown (Fig. 5b). As expected, FOXM1-deficient PEO1 cells display increased sensitivity to carboplatin; however, STL caused no further increase in carboplatin cytotoxic effects in PEO1-shFOXM1 cells, suggesting that FOXM1 is the main mediator of STL effects on cell chemoresistance. At the same time, induction of autophagy by STL was still prominent in FOXM1-deficient cells, indicating that autophagy on its own does not significantly affect chemoresistance.

While platinum-based agents damage DNA directly, 5-FU treatment results in indirect DNA damage due to inhibition of thymidine synthesis [44]. Similar to carboplatin effect, treatment of colorectal cancer cells with 5-FU resulted in FOXM1 upregulation without prominent cell death induction. Combination with STL efficiently prevents 5-FU-induced FOXM1 upregulation and drastically enhances the cytotoxic effects of 5-FU treatment (Fig. 5c). Thus, FOXM1 inhibition by STL can sensitize cancer cells to treatments based on both direct and indirect DNA damage induction.

Taxanes are another class of anticancer drugs that exhibit decreased efficacy against tumor cells with high FOXM1 levels. However, unlike platinum-based compounds or 5-FU, taxanes do not induce prominent DNA damage, affecting mitotic spindle microtubule dynamics instead to disrupt cell division [45]. Accordingly, we did not observe uniform FOXM1 upregulation in prostate cancer cells treated with docetaxel or NSCLC cells treated with paclitaxel (Fig. 5d). Nevertheless, FOXM1 suppression by STL synergized strongly with both docetaxel and paclitaxel, enhancing apoptotic response. This effect indicates that the role of FOXM1 as a chemoresistance inducer is not limited to DNA damage response and can be much more universal.

While caspase-3 cleavage is a common indication of apoptosis induction, cell death should be verified using other methods for better reliability. We therefore additionally verified the cytotoxic effects of STL in combination with other agents using either flow cytometry-based Annexin V assay (Supplementary Fig. 9a–b) or Trypan Blue exclusion assay with direct counting (Supplementary Fig. 9c). The results of these experiments were in strong agreement with trends observed using immunoblotting approach. Subtoxic doses of STL (10 μM) also exerted a clear antiproliferative effect that was associated with moderate enrichment of cells in G1 cell cycle phase (Supplementary Fig. 10).

### RNA-seq data suggest STL selectivity toward FOXM1 regulatory pathway

**Fig. 6:**
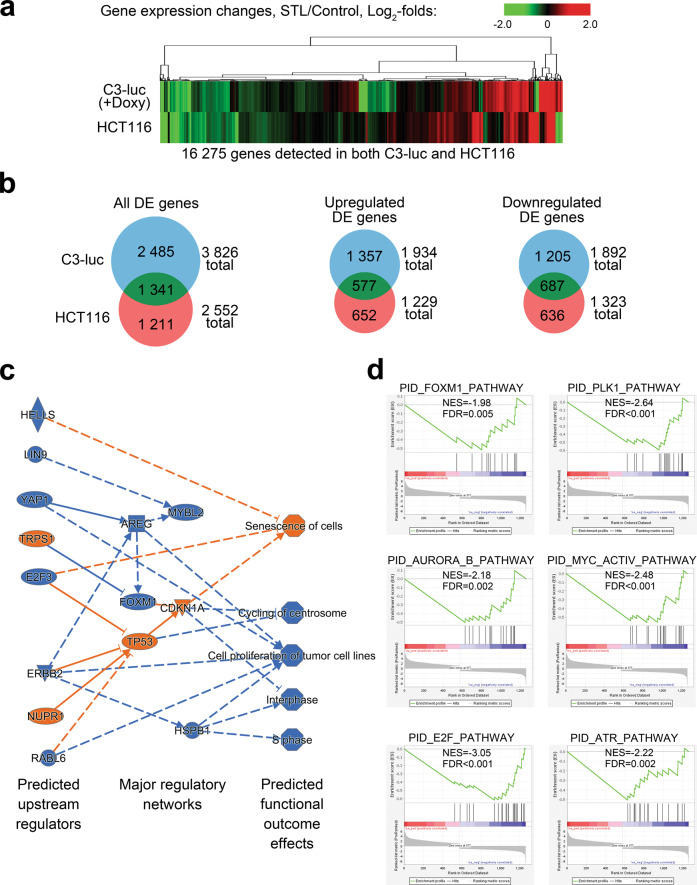
STL-induced transcriptome changes suggest strong antitumor effect mediated through FOXM1-p21–p53 regulatory networks. **a** Heatmap representation of the STL effect on global gene expression changes. HCT116 and doxycycline-stimulated C3-luc cells were treated with 50 μM STL for 24 h. RNA samples obtained from treated cells were subjected to RNA-seq, non-protein-coding genes were excluded from analysis. Data represent the average of two biological replicates for each condition. **b** Venn diagrams representing the numbers of DE genes (all, up- or downregulated) in HCT116 and C3-luc cells. Only genes with significant expression changes (2-fold or higher change, FDR < 0.1) were analyzed. **c** Ingenuity-predicted regulatory network facilitating STL treatment effects in C3-luc cells based on “STL signature” changes. **d** Results of GSEA performed for “STL signature” genes in C3-luc cells.

STL is a novel agent, and its biological effects and targets are not fully characterized yet. We therefore attempted to investigate if it affects any other regulatory pathways besides FOXM1 network. To achieve that, we analyzed gene expression patterns in HCT116 cells and doxycycline-stimulated C3-luc cells treated with STL via full transcriptome RNA-seq (Fig. 6; full processed data on gene expression changes are available in Supplementary Tables 2 and 3). Out of 16 275 protein-coding genes evaluated (Fig. 6a), we identified a set of 1341 genes displaying significant (2-fold or more) DE in both experimental models, with 577 genes being upregulated and 687 genes being repressed in both C3-luc and HCT116 cells (Fig. 6b). We therefore considered the genes displaying codirectional expression changes in both cell lines as the most reliable STL responders, combined them into “STL signature” gene list (1264 genes in total), and subjected to further pathway analysis.

To predict possible regulators and pathways that may be responsible for STL-induced gene expression changes, we performed an integrative analysis of “STL signature” datasets using Ingenuity Pathway Analysis software package. Ingenuity algorithm predicted inhibition of FOXM1 and activation of p53 and p21^Waf1/Cip1^ as central regulatory changes responsible for STL effects on gene expression (Fig. 6c, Supplementary Fig. 11a). Other elements with multiple predicted interactions include AREG and ERBB2, both inhibited, with their downstream effects being partially mediated through FOXM1, p21^Waf1/Cip1^, and p53. These changes in regulation networks are predicted to inhibit cell proliferation at mitosis stage and probably during S phase, while also promoting cell senescence. This prediction is in line with the observed antiproliferative effect of STL and enrichment of cells in G1 phase (Supplementary Fig. 10). All predicted signaling changes and outcome effects strongly suggest that STL treatment should exert clear antitumor effects.

To additionally verify Ingenuity prediction of STL-affected regulation pathways, we performed Gene Set Enrichment Analysis (GSEA) for “STL signature” genes using Pathway Interaction Database (PID) collection of gene signatures [46]. Out of 196 signatures analyzed, six gene sets were significantly enriched in C3-luc cells (Fig. 6d) and seven gene sets—in HCT116 cells (Supplementary Fig. 11b). All gene sets displayed negative normalized enrichment scores, predicting inactivation of the corresponding pathways by STL. It is noteworthy that PLK1, AURKB, and MYC pathways represent the activity of direct FOXM1 downstream effectors, while ATR and BARD1 pathways are responsible for DNA damage response. Moreover, ATR and E2F activity can be modulated by FOXM1 (see “Discussion”), implying that all pathways responsible for STL-induced gene expression changes converge to FOXM1. Taken together, transcriptomic data indicate very high probability of FOXM1 being the main mediator of the effects exerted by STL upon cell gene expression program.

## Discussion

In this paper, we identified a novel chemical compound STL that suppresses FOXM1 activity in a variety of human cancer cell lines (Fig. 1). This drug reduces FOXM1 protein level via a two-step mechanism: it (I) relocates nuclear FOXM1 to the cytoplasm and (II) induces autophagy that facilitates degradation of FOXM1 protein (Figs. 3–4). The exact mechanisms of FOXM1 transport to the cytoplasm and autophagy induction by STL are currently under investigation, but conceptually this is a novel mechanism of FOXM1 suppression.

Previously, we identified several types of FOXM1 inhibitors: thiazole antibiotics/proteasome inhibitors [22, 24, 25] and honokiol [23]. Proteasome inhibitors act through stabilization of HSP70 protein that interacts with FOXM1 and prevents its binding to gene promoters [47]. Honokiol directly binds to FOXM1 protein and inhibits its transactivation potential [23]. In both cases FOXM1 expression is subsequently diminished due to disruption of a positive autoregulation loop [38, 47]. Another group of FOXM1 inhibitors utilizes a different mechanism of action, directly inhibiting FOXM1 DNA-binding capability. Recently, a compound named FDI-6 was identified in a high-throughput screening; it interacts directly with FOXM1 protein, inhibits FOXM1 binding to genomic targets, and therefore suppresses FOXM1 target expression [28]. The authors also provided evidence that FDI-6 selectively targets FOXM1 but not other FOX family proteins. However, FDI-6 does not affect FOXM1 protein level itself [28, 31].

Efficient FOXM1 depletion by STL relies not only on functional inactivation of FOXM1 by its relocalization to the cytoplasm but also on its subsequent autophagic destruction (Figs. 3–4). Recently, the possibility of lysosome-mediated FOXM1 degradation was reported in colon cancer cells. Treatment with DFS lignan resulted in FOXM1 suppression, while BafA1 and CQ were able to prevent this effect [30]. However, the study provides no information about autophagic activity in DFS-treated cells or the mechanism that makes FOXM1 available to autophagosomes. Without these important details, it is impossible to determine if DFS lignan effect is selective to FOXM1 or executed through general nucleophagy. Our research demonstrates for the first time that FOXM1 translocation to the cytoplasm is crucial for its autophagic degradation, suggesting that this process can be selective.

STL-dependent FOXM1 nuclear export and autophagy stimulation seem to be independent from each other: chloroquine treatment does not prevent FOXM1 translocation to the cytoplasm, while FOXM1 retention in the nucleus does not affect autophagy progression (Fig. 4b). Also, lower concentrations of STL efficiently induce autophagy but cannot yet cause prominent reduction of FOXM1 levels (Fig. 3a). We therefore conclude that autophagy induction on its own is not sufficient to promote FOXM1 relocalization, so the latter should be regulated via a separate mechanism. This assumption further supports the idea of STL selectively targeting FOXM1.

Sensitization of resistant cells to chemotherapy, especially to DNA-damaging drugs, is currently the most studied effect of FOXM1 depletion in cancer [8–10, 12]. We expected that STL, being a FOXM1 inhibitor, should decrease drug resistance in human cancer cells when combined with standard chemotherapy drugs. Indeed, we observed clear synergy between STL and three categories of widely used anticancer drugs (carboplatin, 5-FU, and taxanes), each exploiting different mechanisms of anticancer action (Fig. 5). Combining different synergistic drugs is an efficient approach in modern cancer treatment, since it allows not only to improve eradication of cancer cells but also to use chemotherapeutic agents at lower doses, thereby reducing undesired adverse effects. Therefore, STL or its derivatives may turn out useful in clinical practice as support drugs improving the efficacy of existing treatment strategies.

Given that the exact details of STL interactions are unknown, there was a possibility that STL actually regulates chemoresistance through other FOXM1-independent mechanisms. We have demonstrated that FOXM1-deficient cells could not be further sensitized by STL (Fig. 5b), confirming that chemoresistance inhibition is conveyed specifically through FOXM1. However, chemoresistance-independent secondary effects of STL might still be impactful and needed consideration. Transcriptome-based analysis predicted that, besides FOXM1 inhibition, STL may also activate p53- and p21^Waf1/Cip1^-dependent signaling networks (Fig. 6c, Supplementary Fig. 11a). These three proteins are very closely related to each other, forming a single regulatory core. FOXM1 inactivation was reported earlier to increase p53 and p21^Waf1/Cip1^ levels in cancer and nonmalignant cells [48, 49]. Upregulation of p21^Waf1/Cip1^ is facilitated through loss of SCF ubiquitin ligase complex components SKP2 and CKS1B [48]. Alternatively, p53 typically acts as an upstream FOXM1 suppressor [50], but its activation in response to FOXM1 knockdown indicates more complex relations between two molecules [49]. It should be also considered that, even if STL stimulates p53 and p21^Waf1/Cip1^ activity independently of FOXM1, these changes would still result in strong antitumor effects on top of FOXM1-mediated actions. Therefore, such secondary effects should not be regarded as significant disadvantages of STL.

Since Ingenuity predictions are rather speculative, we verified them using GSEA analysis and confirmed the inhibition of FOXM1-related gene signatures but not p53-related effects (Fig. 6d, Supplementary Fig. 11b). Additionally, it revealed the suppression of ATR pathway involved in DNA damage repair [51]. FOXM1 indirectly promotes ATR-dependent signaling through NBS1 [52, 53]. We suppose that impairment of ATR-dependent signaling may contribute to increased vulnerability of FOXM1-deficient cells to chemotherapy, especially to DNA-damaging drugs. Additionally, crucial regulators of E2F pathway, E2F1 and E2F2, can be suppressed upon FOXM1 inhibition [54]. Based on these results, we conclude that most gene expression changes caused by STL are consequent of FOXM1 inhibition, thereby supporting the idea of high specificity of STL toward FOXM1. If the off-target effects of STL are present, then they most likely occur on the post-transcriptional level and would require further detailed investigation.

## Materials and methods

### Cell culture

LNCaP, 22Rv1, and PC3 cell lines (human prostate carcinoma) were provided by Dr. D. J. Vander Griend (University of Illinois at Chicago, Chicago, IL, USA) and Dr. D. G. Tang (Roswell Park Cancer Institute, Buffalo, NY, USA). PEO1, OVCAR3, and OVCAR8 cell lines (human HGSOC) were provided by Dr. J. Burdette (University of Illinois at Chicago). SW480, HCT116, and FET cell lines (human colorectal carcinoma) were provided by Dr. B. Jung (University of Illinois at Chicago). H1703 and A549 cell lines (human NSCLC) were provided by Dr. A. Tyner (University of Illinois at Chicago). C3-luc cell line expressing EGFP-FOXM1 fusion protein controlled by doxycycline-inducible CMV promoter was derived from U2OS cells (human osteosarcoma) as described earlier [25]. Additional details on cell lines used are present in Supplementary Table 1.

LNCaP, 22Rv1, PC3, OVCAR3, H1703, and A549 cells were cultured in RPMI-1640 medium (Thermo Fisher Scientific, Waltham, MA, USA). PEO1 cells were cultured in RPMI-1640 medium with 2 mM sodium pyruvate (Thermo Fisher Scientific). OVCAR8, SW480, HCT116, FET, and C3-luc cells were cultured in DMEM medium with 4.5 g/L glucose, 4 mM l-glutamine, and 1 mM sodium pyruvate (Thermo Fisher Scientific). For all cell lines, the growth media was supplemented with 10% fetal bovine serum (Thermo Fisher Scientific), 100 U/mL penicillin (Lonza, Basel, Switzerland), and 100 μg/mL streptomycin (Lonza). All cell lines were confirmed to be mycoplasma-negative by routine testing using PCR detection and DAPI staining with subsequent evaluation by fluorescent microscopy.

### Chemical compounds and drugs

STL427944 (Vitas-M Laboratory, Hong Kong), actinomycin D (ActD, MilliporeSigma, Burlington, MA, USA), cycloheximide (CHX, MilliporeSigma), bafilomycin A1 (BafA1, AdipoGen Life Sciences, San Diego, CA, USA), bortezomib (APExBIO Technology, Houston, TX, USA), MG132 (Tocris Bioscience, Minneapolis, MN, USA), docetaxel (APExBIO Technology), paclitaxel (APExBIO Technology), and 5-FU (LKT Laboratories, St Paul, MN, USA) were dissolved in DMSO. Doxycycline (LKT Laboratories), chloroquine (CQ, LKT Laboratories), and puromycin (MilliporeSigma) were dissolved in sterile water. Carboplatin (AdipoGen Life Sciences) was dissolved in sterile 5% d-glucose (MilliporeSigma) solution in water. Leptomycin B (LMB, Alfa Aesar, Haverhill, MA, USA) was dissolved in ethanol.

### *In silico* prediction of small-molecule inhibitors of FOXM1 regulatory network

The candidate small molecule inhibitors of FOXM1-regulated signaling network were identified by *in silico* analysis of data from NIH Library of Integrated Network-Based Cellular Signatures (LINCS) L1000 dataset [55]. LINCS L1000 Phase I and Phase II datasets were used (GEO accession IDs: GSE70138 and GSE92742) [56, 57]. DE gene signatures of shRNA-mediated gene knockdowns (more than 3000 individual genes) across most common cell line models (A549, MCF7, VCAP, HA1E, A375, HCC515, and HT29) were compared with transcriptional profiles displayed by the same cell lines upon treatments with a wide range of bioactive compounds. A random forest classification model was trained using data for Food and Drug Administration (FDA)-approved drugs and then used to identify compounds that caused transcriptomic perturbations similar to the chosen genetic disruptions. For each compound, the probability of disrupting the signaling network associated with the protein of interest was evaluated in terms of several attributes, including direct correlation with the transcriptomic signatures of target protein knockdown and indirect correlations with knockdown signatures of other proteins (i.e., “guilt by association” approach suggesting that chemical inhibition acts broadly within a signaling subnetwork), in the subset of four or more cell lines (see [32, 33] for detailed explanation). In the context of protein-signaling networks, a disruption of a physical target due to drug treatment should result in gene expression profiles similar to signatures associated with inhibition of its downstream targets or upstream regulators in the same network.

### Drug treatment of cultured cells

Cells were harvested by trypsinization and seeded into tissue culture dishes to achieve 50% confluency. Cell treatment was performed the next day by aspirating the growth media from the cells and replacing it with growth medium containing selected concentrations of drugs. Control samples were treated with vehicle only, vehicle concentration did not exceed 0.3%. In the endpoint experiments involving treatment with CHX, bortezomib, MG132, BafA1, CQ, or LMB in combination with STL, cells were pretreated with the aforementioned compounds for 1 h before administration of full-treatment drug mixture. In the time-course experiments involving treatment with CHX or CQ in combination with STL, the aforementioned compounds were administered to the cells simultaneously with STL. C3-luc cells with doxycycline-induced expression of EGFP-FOXM1 protein were pretreated with 1 μg/mL doxycycline overnight, and all the following treatments were performed in the presence of 1 μg/mL doxycycline. After the desired periods of time, the cells were immediately harvested, washed once with cold phosphate-buffered saline (PBS), pelleted by centrifugation at 200 g for 5 min, and protein or RNA was purified as described below.

### Stable FOXM1-expression knockdown in PEO1 cells

PEO1 cells were harvested by trypsinization and seeded into 12-well tissue culture plates to achieve 40% confluency. The next day, cells were incubated with MISSION lentiviral particles carrying pLKO.1 vector encoding control nontarget shRNA or shRNA against human FOXM1 transcripts (MilliporeSigma) in the presence of 10 μg/mL polybrene for 24 h. Infected cells were selected by cultivation in the presence of 1.5 μg/mL puromycin for seven days and then cultured as described above.

### Protein immunoblotting

Total protein samples were purified from cells using RIPA lysis buffer (MilliporeSigma) supplemented with Halt protease inhibitor cocktail (Thermo Fisher Scientific), 2 mM sodium orthovanadate (New England Biolabs Inc., Ipswich, MA, USA), and 5 mM sodium fluoride (MilliporeSigma) according to the manufacturer’s protocol. Protein concentrations were estimated using Bio-Rad Protein Assay (Bio-Rad, Hercules, CA, USA). About 15–30 μg of total protein were mixed with Laemmli sample buffer (Bio-Rad) with β-mercaptoethanol (Bio-Rad, final concentration 2.5%), heated at 98 °C for 10 min, and separated in hand-cast 12% SDS–polyacrylamide gels or 12% Mini-PROTEAN TGX precast gels (Bio-Rad). After the electrophoretic separation, the proteins were transferred to Immobilon-P^sq^ PVDF membrane (MilliporeSigma). Membranes were washed with tris-buffered saline (TBS, Alfa Aesar) for 10 min, blocked with 5% bovine serum albumin (BSA, MilliporeSigma) in TBS with 0.1% Tween-20 (TBST, Thermo Fisher Scientific), and probed with the primary antibodies diluted in 5% BSA in TBST overnight at 4 °C (see Supplementary Table 4 for the list of antibodies used). Membranes were washed with TBST three times, 10 min each, and probed with HRP-conjugated secondary antibodies diluted in 5% skim milk (Research Products International, Mt Prospect, IL, USA) in TBST for 1 h at room temperature. Membranes were washed with TBST three times, 10 min each, protein bands were developed using SuperSignal West Pico PLUS substrate (Thermo Fisher Scientific) and detected using ChemiDoc MP System (Bio-Rad). For each immunoblot image in the paper, molecular weights of protein markers are indicated on the right.

### RT-qPCR analysis of gene expression

Total RNA was isolated from cells using TRIzol reagent (Thermo Fisher Scientific) and the PureLink RNA Mini Kit (Thermo Fisher Scientific) with additional on-column DNAse treatment according to the manufacturer’s instructions. RNA samples were quantified using NanoDrop One Spectrophotometer (Thermo Fisher Scientific). Reverse transcription was performed using High-Capacity cDNA Reverse Transcription Kit with RNase Inhibitor (Thermo Fisher Scientific), 500 ng of total RNA was used per reaction. Quantitative PCR analysis of gene expression levels was performed in ViiA 7 Real-Time PCR System (Thermo Fisher Scientific) using PowerUp SYBR Green Master Mix (Thermo Fisher Scientific) and primers listed in Supplementary Table 5. Amplification was performed according to the manufacturer’s Fast Mode recommendations for 35 cycles, reaction specificity was checked by melt-curve analysis and agarose electrophoresis. Reaction efficiency was evaluated using standard curve approach and was within 95–105% for all primers. Transcript abundance was estimated using Pfaffl’s method [58], 18 S rRNA and *TBP* transcripts were used as references for normalization.

### Vital fluorescent staining of lysosomes

Cells were seeded into 35-mm cell culture dishes with glass bottom (MatTek Life Sciences, 200 Homer Ave, Ashland, M, USA) to achieve 30–40% confluency. The next day, the cells were treated with drugs as described above. After 12 h of treatment, LysoView 540 dye (Biotium, Inc., Fremont, CA, USA) was added to the treatment media up to 1x working concentration and cells were incubated at 37 °C in a CO_2_ incubator for 2 h. Cell imaging was performed with LSM 710 confocal microscope (Zeiss, Oberkochen, Germany) using excitation wavelength of 561 nm for LysoView 540 fluorescence or differential interference contrast for cell morphology. Digital images were processed and exported using ZEN 3.2 Blue Edition software package (Zeiss).

### Immunofluorescent staining of cell proteins

Cells were seeded into Lab-Tek™ II Chamber Slides (Thermo Fisher Scientific) to achieve 30–40% confluency. The next day, the cells were treated with drugs as described above. After treatment, cells were briefly washed with cold PBS, fixed with 100% methanol at −20 °C for 20 min, and washed with cold PBS three times. Cells were blocked with 2% BSA in PBS with 0.1% Tween-20 (PBST, Thermo Fisher Scientific) for 1 h at room temperature and stained with primary antibodies against LC3A/B (Cell Signaling Technology, D3U4C, 1:300) diluted in 2% BSA in PBST overnight at 4 °C. Cells were washed three times with cold PBST and stained with secondary anti-rabbit antibodies conjugated with Alexa Fluor 594 (Jackson Immunoresearch, 711-585-152, 1:1000) diluted in 2% BSA in PBST for 1 h at room temperature in the dark. After immunostaining, the cells were counterstained with 0.5 μg/mL DAPI (R&D Systems, Minneapolis, MN, USA) diluted in PBST for 10 min at room temperature and washed three times with PBST. Chambers were removed from the slides and coverslips were mounted on slides using ProLong Diamond Antifade mountant (Thermo Fisher Scientific). Slides with mounted coverslips were kept at room temperature in the dark overnight and then stored at 4 °C. Cell imaging was performed with LSM 710 confocal microscope (Zeiss, Oberkochen, Germany) using excitation wavelengths of 405 nm for DAPI, 488 nm for EGFP, and 561 nm for Alexa Fluor 594 detection. Images were taken by an operator blinded to treatment groups and not instructed to focus on specific features. Digital images were processed and exported using ZEN 3.2 Blue Edition software package (Zeiss).

### Protein colocalization analysis of immunofluorescent microscopic images

Digital images obtained via confocal microscopy (24-bit TIFF, RGB color) were split into red, green, and blue channels using ImageJ software package [59]. The images for each channel were processed using JACoP plugin [60], signal threshold values were optimized for most efficient separation of signal from background and kept constant for all analyzed images. Manders’ overlap coefficients were calculated, where applicable, for fractions of green pixels (EGFP-FOXM1) being colocalized with red pixels (LC3 staining, mostly cytoplasmic) or with blue pixels (DAPI staining, nuclear). Twelve individual cells taken from different fields of view were analyzed for each treatment condition.

### Annexin V-based detection of apoptotic cells

Cells were seeded into 60 mm cell culture dishes (Thermo Fisher Scientific) to achieve 30-40% confluency. The next day, the cells were treated with drugs as described above. After treatment, cells were harvested by mild trypsinization, washed twice with ice-cold PBS, and 500 000 cells were resuspended in 100 μL of Annexin V Binding Buffer (BD Biosciences, San Jose, CA, USA). Cells were stained by incubating with 5 μL of APC-conjugated Annexin V recombinant protein (Thermo Fisher Scientific) for 15 min in the dark, pelleted by centrifugation at 200 g for 5 min, and resuspended in 300 μL of Annexin V Binding Buffer containing 0.1 μg/mL DAPI (R&D Systems). Samples were analyzed using CytoFLEX flow cytometer and CytExpert software (Beckman Coulter, Brea, CA, USA).

### Trypan Blue exclusion cell viability assay

Cells were seeded into 12-well cell culture plates (Thermo Fisher Scientific) at 50 000 cells/well. The next day the cells were treated with drugs as described above for 72 h. After treatment cells were harvested by mild trypsinization, washed twice with ice-cold PBS, and resuspended in 200 μL of Hanks’ Balanced Salt Solution (HBSS) without Ca^2+^ and Mg^2+^ (Lonza). Numbers of viable and dead cells were assessed by direct counting using a hemocytometer in the presence of 0.4% Trypan Blue.

### Cell cycle assay

Cells were seeded into 60-mm cell culture dishes (Thermo Fisher Scientific) to achieve 30–40% confluency. The next day, the cells were treated with drugs as described above. After treatment, cells were harvested by mild trypsinization, resuspended in 300 μL of ice-cold PBS, and fixed by the addition of 0.7 mL of ice-cold 70% ethanol in a dropwise manner with constant mixing. After addition of ethanol, samples were stored at −70 °C overnight. Fixed cell samples were washed with ice-cold ethanol twice and stained with 1 µg/mL DAPI (R&D Systems). Stained samples were analyzed using CytoFLEX flow cytometer and CytExpert software (Beckman Coulter), at least 25 000 qualifying events were detected in each evaluated sample.

### Full-transcriptome RNA-seq

RNA samples were analyzed for integrity using Agilent 4200 TapeStation (Agilent Technologies, Santa Clara, CA, USA). The levels of the remaining DNA were checked using Qubit fluorometer (Thermo Fisher Scientific). DNA amounts did not exceed 10% of the total amount of nucleic acid.

Sequencing libraries for Illumina sequencing were prepared in one batch in a 96-well plate using Stranded CORALL total RNAseq library prep kit with RiboCop HMR rRNA Depletion Kit (Lexogen, Vienna, Austria). In brief, 260–660 nanograms of total RNA were used for the first rRNA depletion step, then followed by library generation initiated with random oligonucleotide primer hybridization and reverse transcription. No prior RNA fragmentation was done, as the insert size was determined by proprietary size-restricting method. Next, the 3′ ends of first-strand cDNA fragments were ligated with a linker containing Illumina-compatible P5 sequences and unique molecular identifiers. During the following steps of second-strand cDNA synthesis and dual-strand cDNA amplification, i7 and i5 indices as well as complete adapter sequences required for cluster generation were added. A number of PCR amplification cycles was 12, as determined by qPCR using a small preamplification library aliquot for each individual sample.

The final amplified libraries were purified, quantified, and average fragment sizes confirmed to be 330 bp by gel electrophoresis using Agilent 4200 TapeStation (Agilent Technologies). The concentration of the final library pool was confirmed by qPCR and then subjected to test sequencing in order to check sequencing efficiencies and adjust accordingly the proportions of individual libraries. Sequencing was carried out on NovaSeq 6000, S4 flowcell (Illumina, San Diego, CA, USA), approximately 30 M 2 × 150-bp clusters per sample.

### Bioinformatical analysis of RNA-seq data

Analysis of raw RNA-seq data was performed by Research Informatics Core at the University of Illinois at Chicago. Raw reads were aligned to the human hg38 reference genome in a splice-aware manner using the STAR aligner [61]. ENSEMBL gene and transcript annotations, including noncoding RNAs, were used. Expression levels of features, i.e., genes and noncoding RNAs, were quantified using FeatureCounts as raw read counts [62].

DE statistics (fold change and *p*-value) were computed using edgeR on raw expression counts obtained from quantification [63, 64]. Raw expression counts were normalized within edgeR using TMM normalization. Nominal p-values were adjusted for multiple testing using the false-discovery rate (FDR) correction of Benjamini and Hochberg [65]. Significant genes were determined based on fold changes lower than 0.5 and higher than 2.0, FDR threshold of 10% (*q*-value < 0.1) in the multigroup comparison. Processed data on gene expression levels are provided in Supplementary Table 2 (C3-luc cells) and Supplementary Table 3 (HCT116 cells).

Regulatory pathway analysis was performed in Ingenuity Pathway Analysis software package (QIAGEN, Hilden, Germany) and GSEA software (University of California San Diego and Broad Institute, USA [66, 67]), using “STL signature” gene list (see “Results”). Input data contained log_2_-fold-change values, *p*-values, and FDR *q*-values for each gene. For Ingenuity analysis, data were analyzed using direct and indirect interactions reported for human samples. Correlations derived from machine-based learning were not considered. For GSEA analysis, GSEAPreranked algorithm was used to analyze data using PID collection of canonical pathway gene signatures [46] with a number of permutations set to 1000. Pathways with FDR < 0.05 were considered significantly enriched.

### Statistical analysis

At least three independent biological replicates were used for all experiments describing cell treatment with drugs, excluding RNA-seq, where two biological replicates were used, and confirmational Annexin V assay and cell cycle assay, where only one experiment was performed. RT-qPCR experiments were performed with two technical replicates for each biological replicate. For immunoblot experiments, the images shown in the paper represent the results that were consistent across several independent experiments. The statistical tests used in each experiment are described in the corresponding figure and table legends. Statistical significance was accepted with *p* < 0.05. Statistical analysis was performed in OriginPro 2016 software (OriginLab Corporation, Northampton, MA, USA). Plots were generated using GraphPad Prism 6 software (GraphPad Software, San Diego, CA, USA).

Clustering of RNA sequencing data and heatmap plot generation were performed in Morpheus software (https://software.broadinstitute.org/morpheus, Broad Institute, USA) using Euclidean metrics and complete linkage settings for clustering.

## Supplementary information

Suppl figures

Suppl tables

Suppl table 2

Suppl table 3

## Data Availability

LINCS L1000 Phase I and Phase II datasets used for chemical compound screening are available from Gene Expression Omnibus (accession IDs: GSE70138 and GSE92742). Raw RNA-seq data on gene expression levels in C3-luc and HCT116 cells treated with STL are available from Gene Expression Omnibus (accession ID GSE162826). Processed RNA-seq data on gene expression levels in C3-luc and HCT116 cells treated with STL are included in this paper as Supplementary Table 2 (C3-luc cells) and Supplementary Table 3 (HCT116 cells).
