# Supplementary material for: Novel FOXM1 inhibitor identified via gene network analysis induces autophagic FOXM1 degradation to overcome chemoresistance of human cancer cells
Source: Cell Death Dis. 2021 Jul 14;12(7):704. doi: 10.1038/s41419-021-03978-0 (PMC8280155; doi:10.1038/s41419-021-03978-0)
Supplement: Supplementary file 1 — Suppl figures [file 41419_2021_3978_MOESM1_ESM.pdf]

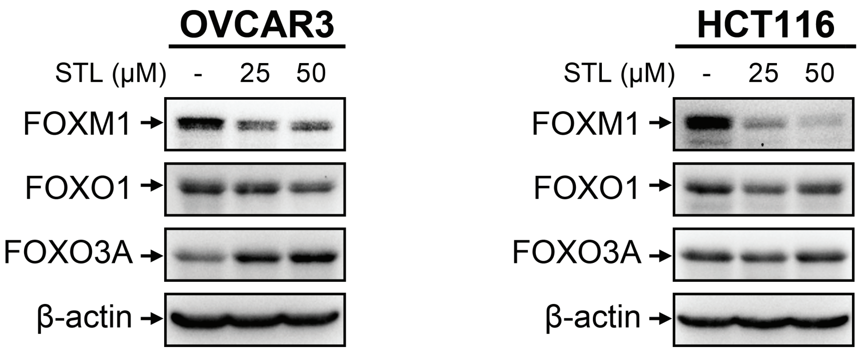

Supplementary Figure 1. STL treatment suppresses FOXM1 but not other FOX family proteins. OVCAR3 and HCT116 cell lines were treated with indicated concentrations of STL for 24 hours. Total protein samples obtained from treated cells were analyzed for FOXM1, FOXO1 and FOXO3A protein levels via immunoblotting, β-actin was used as internal loading control.

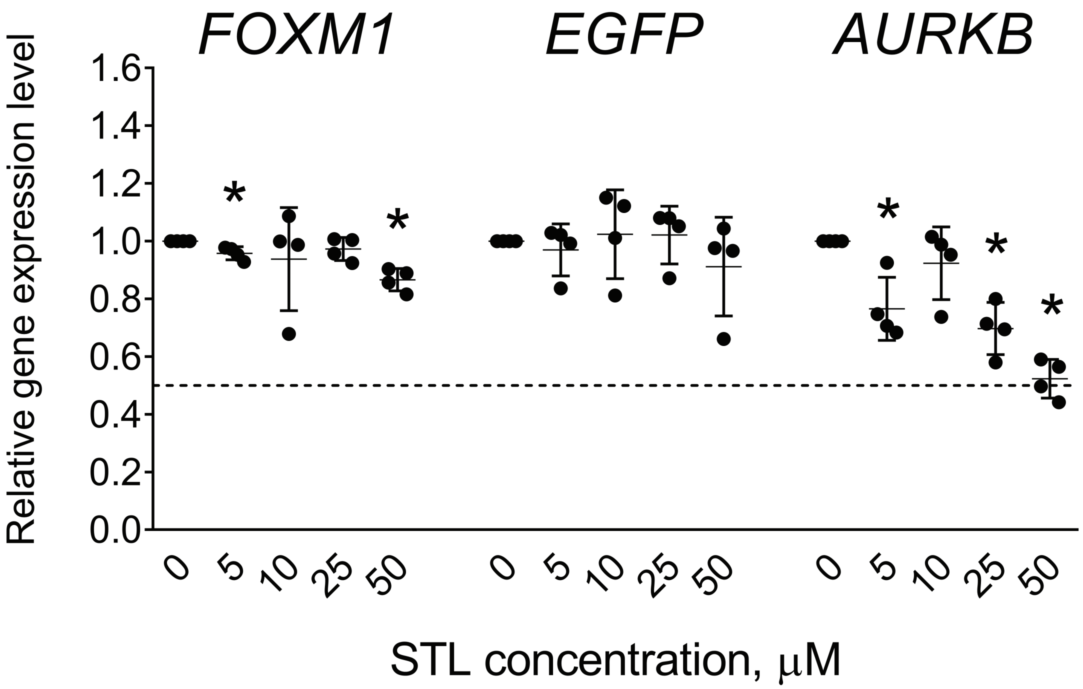

Supplementary Figure 2. STL exerts dose-dependent suppressive effect on FOXM1 downstream target *AURKB*. Doxycycline-stimulated C3-luc cells were treated with indicated concentrations of STL for 24 hours. Total RNA samples were analyzed for *FOXM1*, *EGFP* and *AURKB* transcript levels via RT-qPCR, *TBP* was used as a reference transcript. Data are presented as mean ± S.D. and individual datapoints, N=4, * – exact p=0.02857 (Mann-Whitney U test, two-tailed).

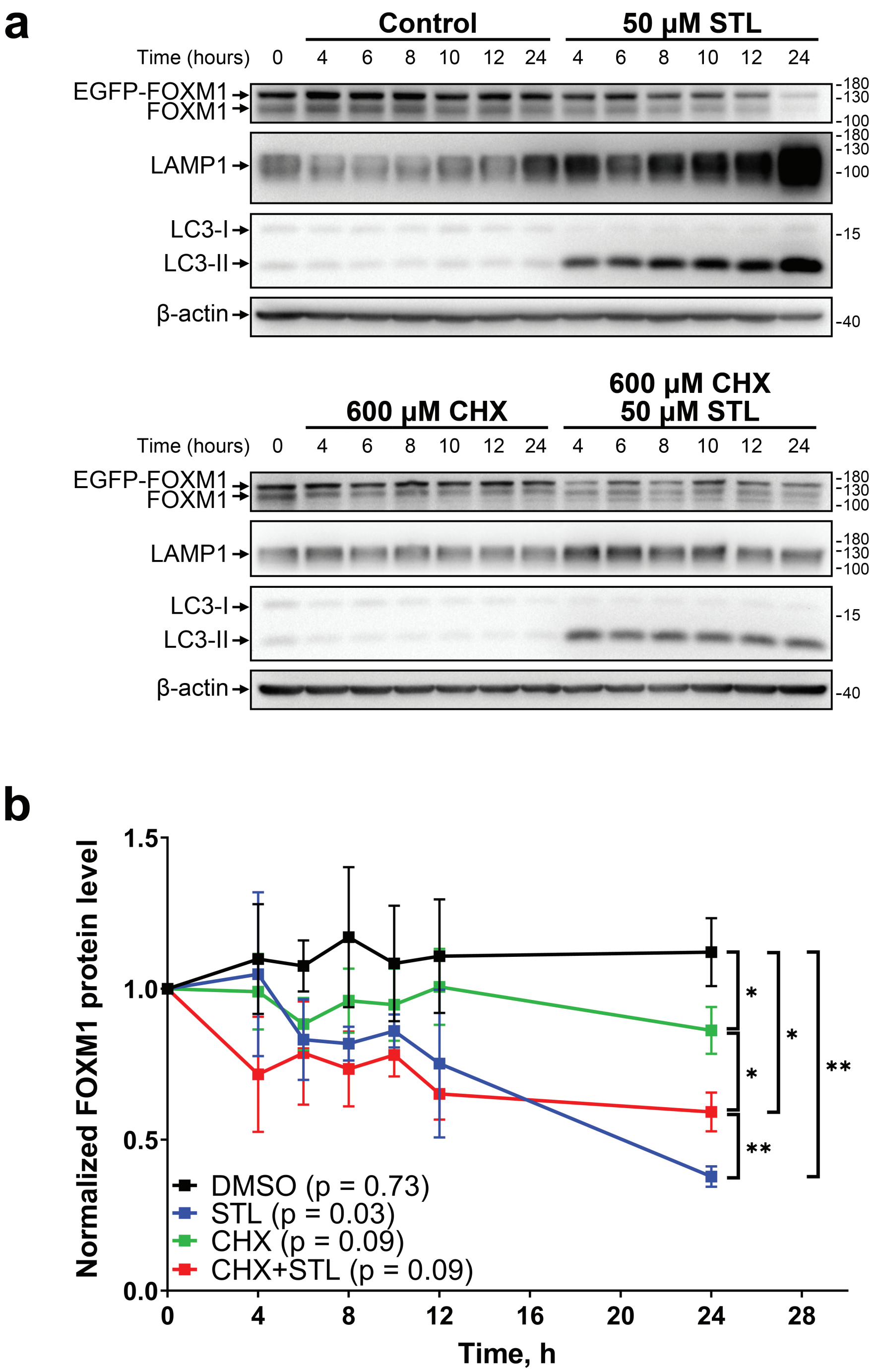

Supplementary Figure 3. STL treatment results in FOXM1 protein degradation over time that is prevented by CHX. a Doxycycline-stimulated C3-luc cells were treated with indicated concentrations of STL and CHX (added simultaneously) for 4, 6, 8, 10, 12, or 24 hours. Total protein samples were analyzed via immunoblotting for FOXM1, LAMP1, and LC3 expression, β-actin was used as an internal loading control. **b** Time profile of FOXM1 protein expression in doxycycline-stimulated C3-luc cells treated with STL and CHX. Densitometry-estimated FOXM1 levels were normalized to corresponding β-actin levels and "0 hours" sample, data are presented as mean ± S.D., N=4-6 (for different time points). * – $p<0.05$, ** – $p<0.01$ (Mann-Whitney U test, two-tailed), p-values in the legend of panel **b** represent the significance of changes over time determined using Friedman's ANOVA test.

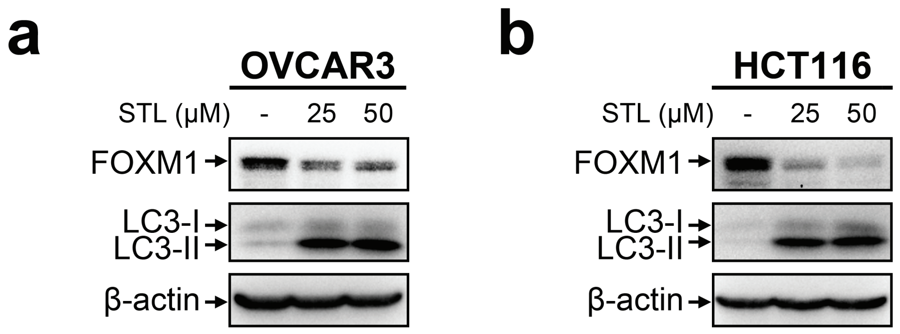

Supplementary Figure 4. STL induces autophagy in cancer cell lines with endogenous FOXM1 only. OVCAR3 (**a**) and HCT116 (**b**) cells were treated with indicated concentrations of STL for 24 hours. Total protein samples were analyzed via immunoblotting for FOXM1 and LC3 expression, β-actin was used as an internal loading control.

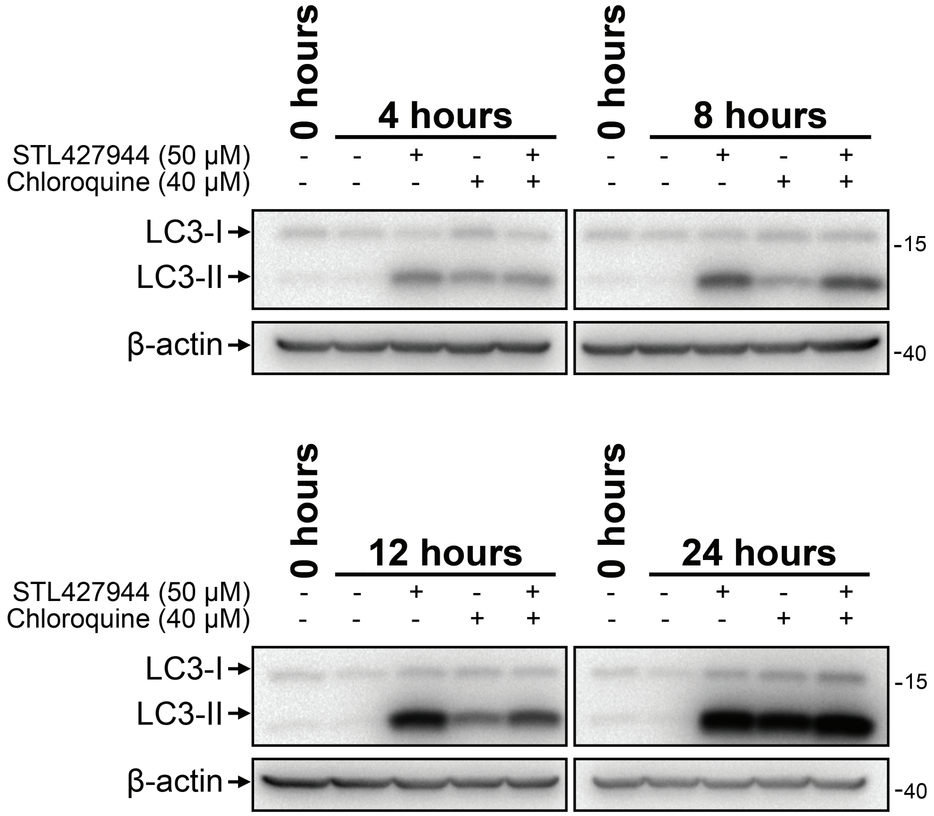

Supplementary Figure 5. STL causes faster LC3-II accumulation than inhibition of autophagosome degradation. Doxycycline-stimulated C3-luc cells were treated with indicated concentrations of STL and CQ (added simultaneously) for 4, 8, 12, or 24 hours. Total protein samples were analyzed via immunoblotting for LC3 expression, β-actin was used as an internal loading control.

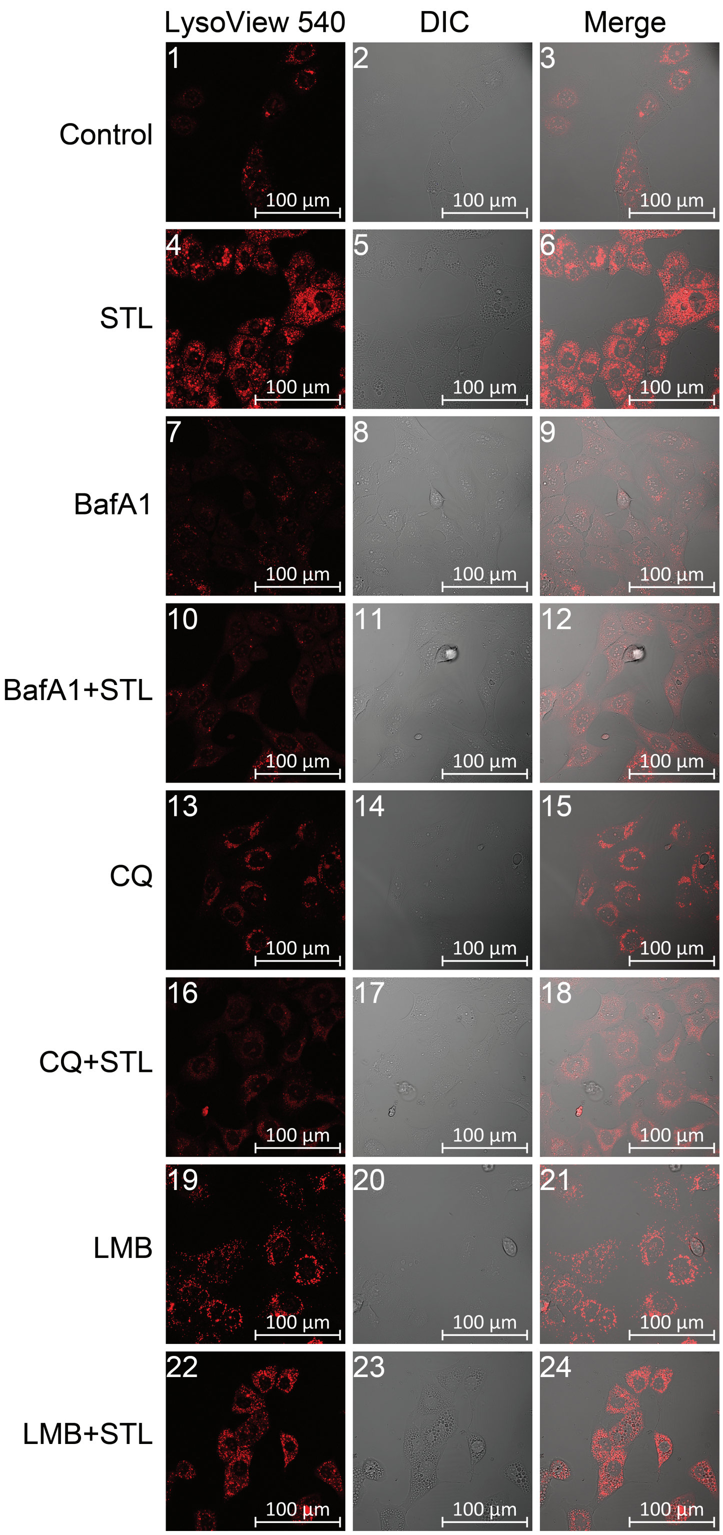

Supplementary Figure 6. STL promotes active lysosome formation. Doxycycline-stimulated C3-luc cells expressing EGFP-FOXM1 fusion protein were treated with vehicle ("Control", panels 1-3), 50 M STL ("STL", panels 4-6), 25 nM Baf1A ("Baf1A", panels 7-9), 40 µM CQ ("CQ", panels 13-15), 25 nM LMB ("LMB", panels 19-21), or indicated drug combinations (panels 10-12, 16-18, 22-24) for 12 hours. Lysosomes were stained with vital LysoView 540 dye (red), cell morphology was analyzed using differential interference contrast (DIC) microscopy.

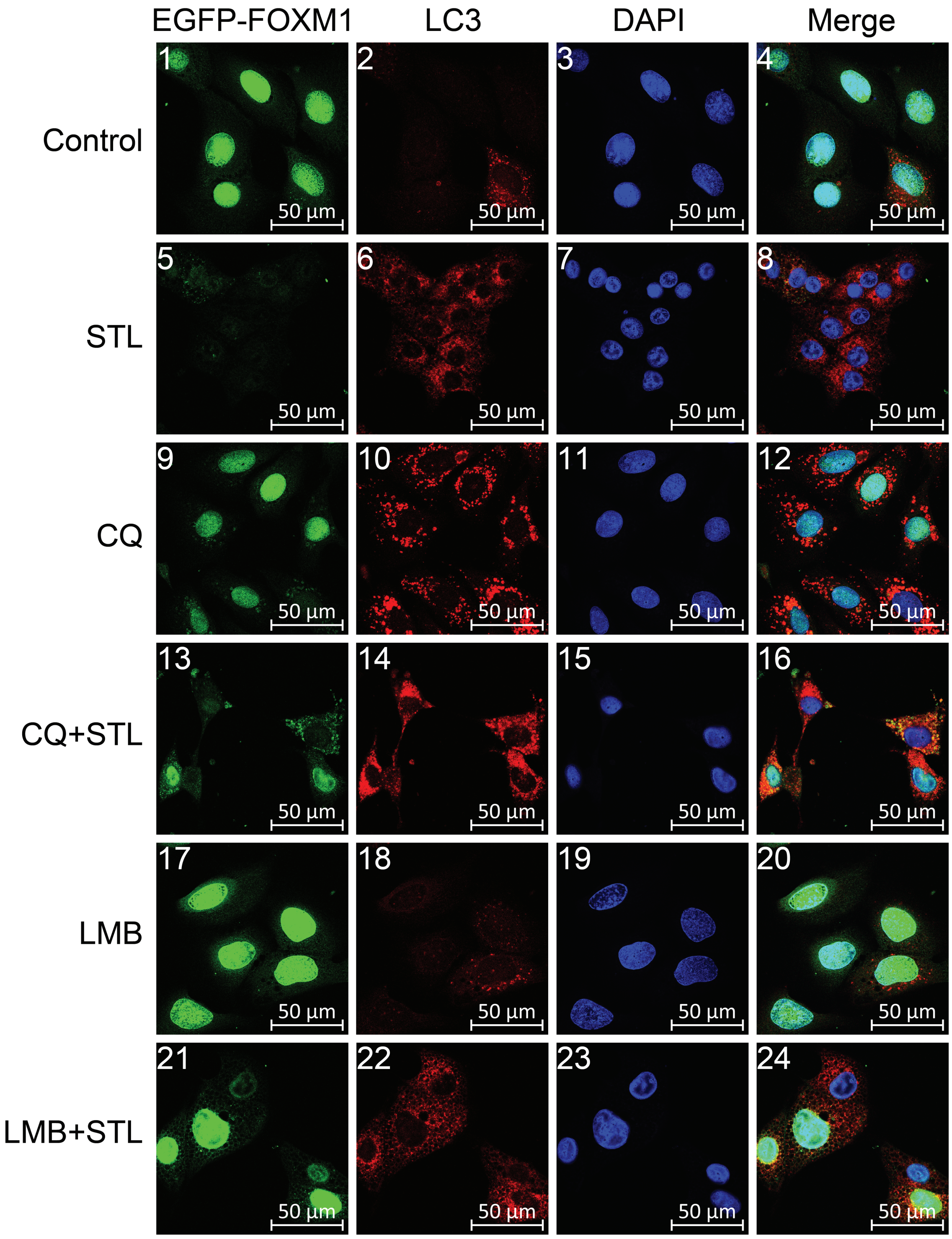

Supplementary Figure 7. STL promotes FOXM1 translocation from the nucleus to the cytoplasmic autophagosomes. Doxycycline-stimulated C3-luc were treated with vehicle ("Control", panels 1-4), 50 M STL ("STL", panels 5-8), 40 µM CQ ("CQ", panels 9-12), 25 nM LMB ("LMB", panels 17-20), or indicated drug combinations (panels 13-16, 21-24) for 24 hours. Cells were stained for LC3 protein, nuclei were counterstained with DAPI. EGFP-FOXM1 (green), LC3 (red) and DAPI (blue) fluorescence was analyzed using confocal laser scanning microscopy.

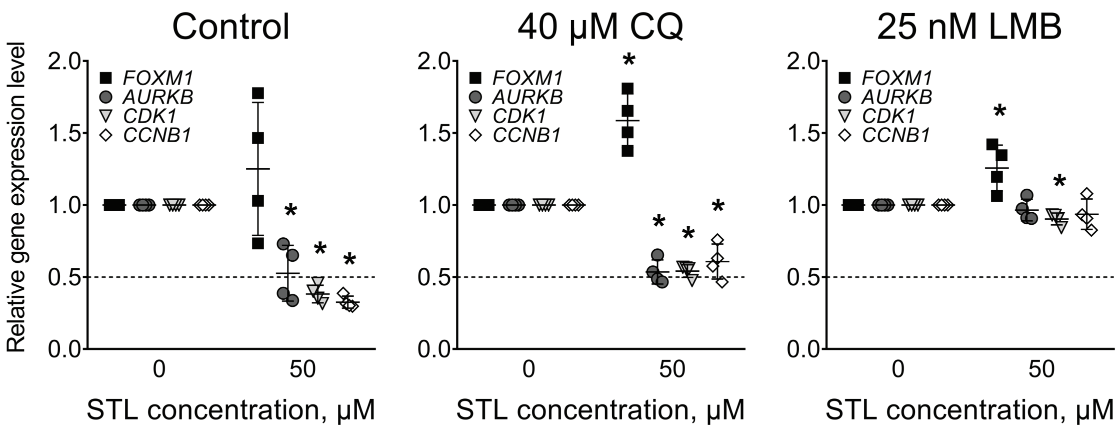

Supplementary Figure 8. FOXM1 relocalization to cytoplasm by STL results in its functional inactivation. Doxycycline-stimulated C3-luc cells were treated with indicated concentrations of STL, CQ, and LMB for 24 hours. Total RNA samples were analyzed for *FOXM1*, *AURKB*, *CDK1*, and *CCNB1* transcript levels via RT-qPCR, *TBP* was used as a reference transcript. Data are presented as mean ± S.D. and individual datapoints, N=4, * – exact p=0.02857 (Mann-Whitney U test, two-tailed).

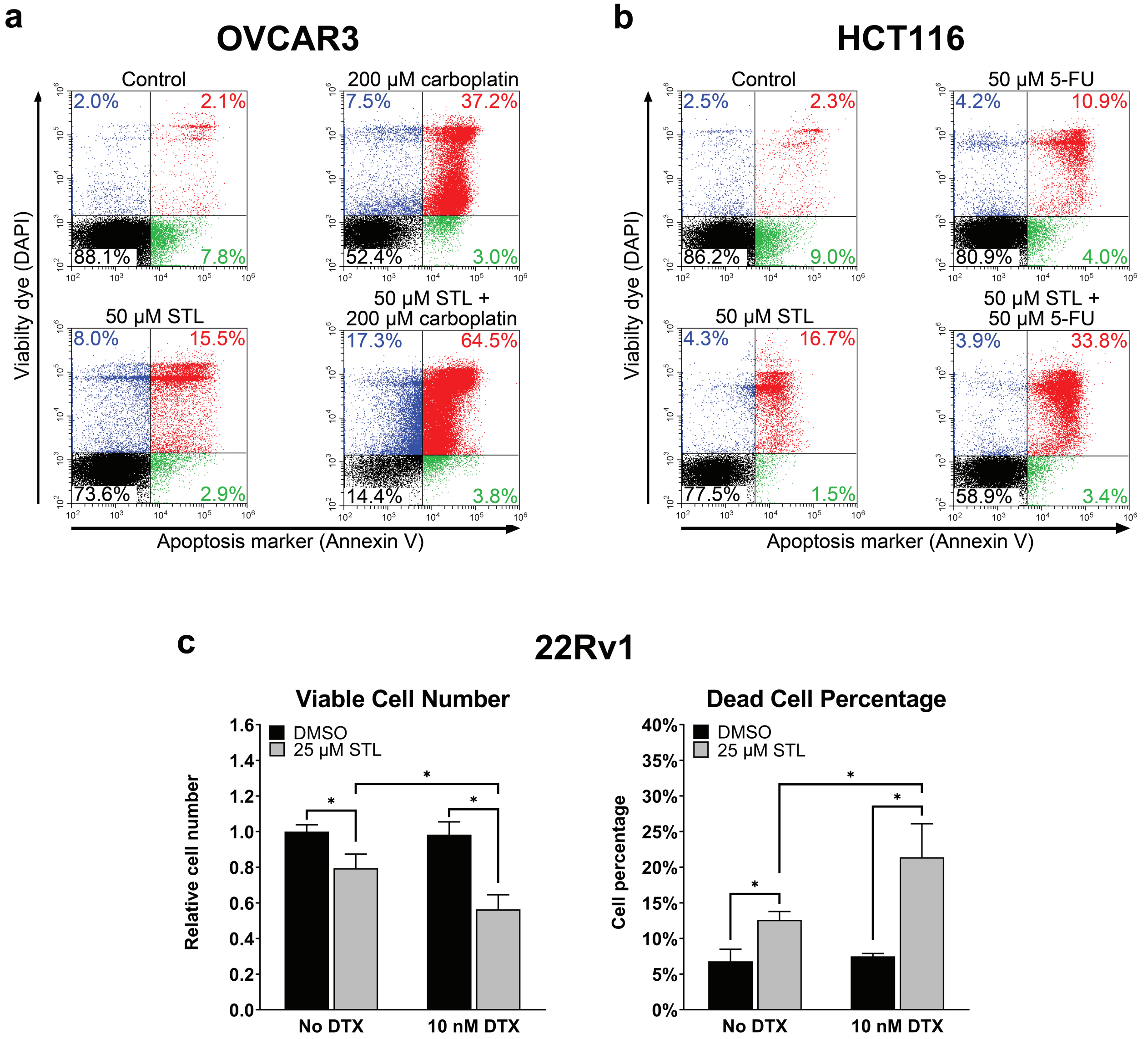

Supplementary Figure 9. STL enhances cytotoxic effect of conventional chemotherapeutic drugs. a OVCAR3 cells were treated with indicated concentrations of STL and carboplatin for 48 hours. **b** HCT116 cells were treated with indicated concentrations of STL and 5-FU for 24 hours. Flow cytometry-based Annexin V assay was performed to identify viable (DAPI^{low}/Annexin V^{low}, black), early apoptotic (DAPI^{low}/Annexin V^{high}, green), early necrotic (DAPI^{high}/Annexin V^{low}, blue) and dead (DAPI^{high}/Annexin V^{high}, red) cells. **c** 22Rv1 cells were treated with indicated concentrations of STL and DTX for 24 hours. The numbers of viable and dead cells were counted in presence of Trypan Blue and normalized to “DMSO, No DTX” sample. Data are presented as mean + S.D., N=4, * – $p<0.05$ (Mann-Whitney U test, two-tailed).

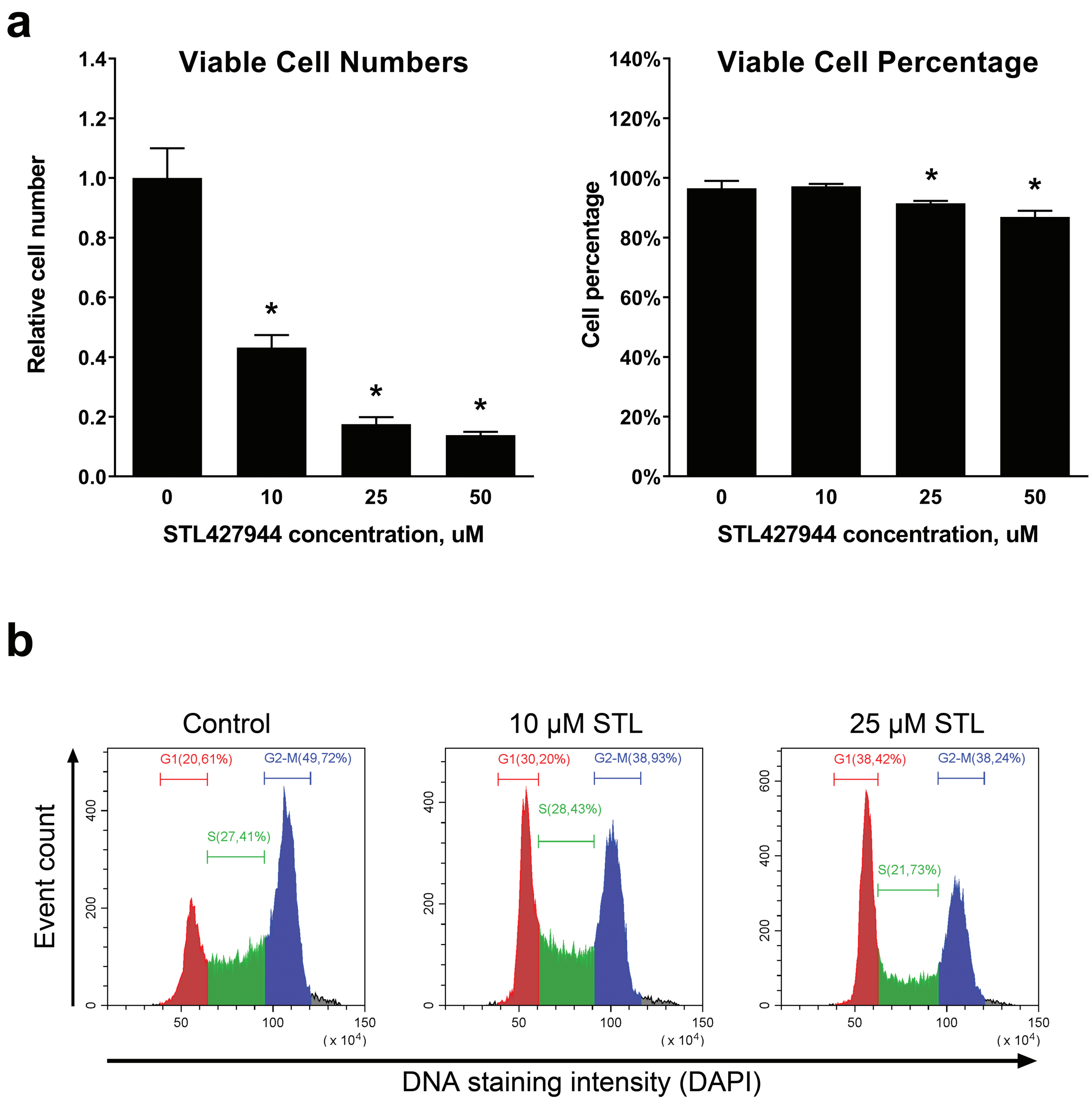

Supplementary Figure 10. Sub-toxic STL concentrations inhibit cell proliferation by arresting cell cycle in G1 phase. a OVCAR3 cells were treated with indicated concentrations of STL for 72 hours. The numbers of viable and dead cells were counted in presence of Trypan Blue and normalized to “0 μM” sample. Data are presented as mean + S.D., N=4, * – $p<0.05$ (Mann-Whitney U test, two-tailed). **b** OVCAR3 cells were treated with indicated concentrations of STL for 24 hours, fixed and stained with DAPI. Flow cytometry-based cell cycle assay was performed to estimate fractions of cells in G1, S, and G2/M phases.

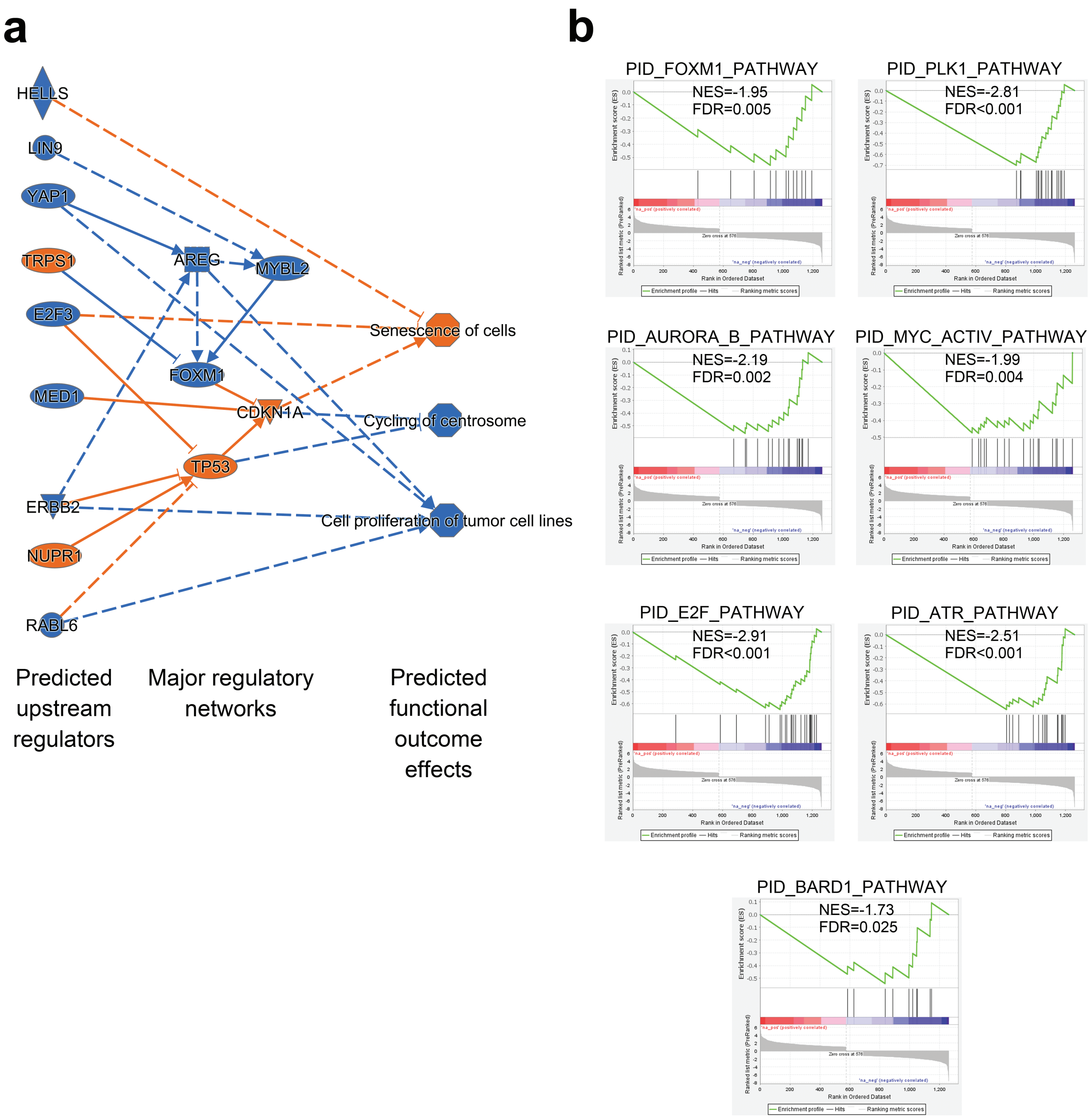

Supplementary Figure 11. Pathway analysis of STL-induced transcriptome changes in HCT116 cells. a IPA-predicted regulatory network facilitating STL treatment effects in HCT116 cells. **b** Results of GSEA performed for “STL signature” genes in HCT116 cells.
