# Supplementary material for: Novel FOXM1 inhibitor identified via gene network analysis induces autophagic FOXM1 degradation to overcome chemoresistance of human cancer cells
Source: Cell Death Dis. 2021 Jul 14;12(7):704. doi: 10.1038/s41419-021-03978-0 (PMC8280155; doi:10.1038/s41419-021-03978-0)
Supplement: Supplementary file 2 — Suppl tables [file 41419_2021_3978_MOESM2_ESM.pdf]

Supplementary Table 1. Human cancer cell lines used in the study.

Cell line	Tissue of origin	Cancer type	FDA-approved drugs	Additional features
LNCaP	Prostate	Prostate carcinoma	Docetaxel, enzalutamide, olaparib	Metastatic tumor
22Rv1		Prostate carcinoma		
PC3		Prostate carcinoma		Metastatic tumor, grade IV
PEO1	Ovary	High grade serous adenocarcinoma	Cisplatin, carboplatin, oxaliplatin, paclitaxel, olaparib	Platinum-sensitive
OVCAR3		High grade serous adenocarcinoma		Platinum-sensitive
OVCAR8		High grade serous adenocarcinoma		Platinum-resistant
SW480	Colon	Colorectal adenocarcinoma	5-FU, bevacizumab, irinotecan, oxaliplatin	Activating RAS mutation
FET		Colon carcinoma		Activating RAS mutation
HCT116		Colorectal carcinoma		Activating RAS mutation
A549	Lung	Lung adenocarcinoma	Carboplatin, docetaxel, paclitaxel, bevacizumab, erlotinib	
H1703		Non-small cell lung cancer		
U2OS	Bone	Osteosarcoma	Doxorubicin, dactinomycin	Was used to generate C3-luc cells expressing EGFP-FOXM1 fusion protein

Supplementary Table 4. List of antibodies used for immunoblotting.

Protein target	Antibody supplier,	Antibody host and clone/ID	Antibody working dilution
FOXM1	Abcam, USA	Rabbit monoclonal, EPR17379	1:1,000
β-actin	MilliporeSigma, USA	Mouse monoclonal, AC-15	1:20,000
GFP	Thermo Fisher Scientific, USA	Mouse monoclonal, GF28R	1:500
MCL1	Cell Signaling Technology, USA	Rabbit monoclonal, D5V5L	1:1,000
NPM	Santa Cruz Biotechnology	Mouse monoclonal, FC82291	1:3,000
Cleaved caspase-3	Cell Signaling Technology, USA	Rabbit monoclonal, 5A1E	1:1,000
LC3A/B	Cell Signaling Technology, USA	Rabbit monoclonal, D3U4C	1:3,000
LAMP1	Cell Signaling Technology, USA	Rabbit monoclonal, D2D11	1:3,000
anti-rabbit IgG, HRP-conjugated	Jackson Immunoresearch, USA	Alpaca polyclonal, 611-035-215	1:10,000
anti-mouse IgG, HRP-conjugated	Jackson Immunoresearch, USA	Donkey polyclonal, 715-035-150	1:10,000

Supplementary Table 5. List of primers used in RT-qPCR analysis.

Gene	Forward primer	Reverse primer
18S rRNA	GACTCAACACGGGAAACCTC	TCGCTCCACCAACTAAGAAC
TBP	GAGAGTTCTGGGATTGTACC	GGATTATATTCGGCGTTTCG
FOXM1	CGAAAGATGAGTTCTGATGGAC	TCCTCTCAGTGCTGTTGATG
EGFP	AGAAGAACGGCATCAAGGTG	GTGCTCAGGTAGTGGTTGTC
MCL1	TGGCAAGAGGATTATGGCTAAC	TTACACAGGTCACTGGCATTC
AURKB	AAGGGAGAGCTGAAGATTGC	TGAGCAGTTTGGAGATGAGG
CCNB1	TTTCGCCTGAGCCTATTTTG	CCATCTTCTGCATCCACATC
CDK1	GATCTACCATACCCATTGACTAAC	ATGGCTACCACTTGACCTG
